# Multiple isoforms of phospho*enol*pyruvate carboxylase in the Orchidaceae (subtribe Oncidiinae): implications for the evolution of crassulacean acid metabolism

**DOI:** 10.1093/jxb/eru234

**Published:** 2014-06-09

**Authors:** Katia Silvera, Klaus Winter, B. Leticia Rodriguez, Rebecca L. Albion, John C. Cushman

**Affiliations:** ^1^Smithsonian Tropical Research Institute, PO Box 0843-03092, Balboa, Ancon, Republic of Panama; ^2^Department of Biochemistry & Molecular Biology, MS330, University of Nevada, Reno, NV 89557-0330, USA

**Keywords:** Crassulacean acid metabolism, gene duplication, Orchidaceae, Oncidiinae, phosphoenolpyruvate carboxylase, photosynthesis.

## Abstract

Phospho*enol*pyruvate carboxylase (PEPC) catalyses the initial fixation of atmospheric CO_2_ into oxaloacetate and subsequently malate. Nocturnal accumulation of malic acid within the vacuole of photosynthetic cells is a typical feature of plants that perform crassulacean acid metabolism (CAM). PEPC is a ubiquitous plant enzyme encoded by a small gene family, and each member encodes an isoform with specialized function. CAM-specific PEPC isoforms probably evolved from ancestral non-photosynthetic isoforms by gene duplication events and subsequent acquisition of transcriptional control elements that mediate increased leaf-specific or photosynthetic-tissue-specific mRNA expression. To understand the patterns of functional diversification related to the expression of CAM, *ppc* gene families and photosynthetic patterns were characterized in 11 closely related orchid species from the subtribe Oncidiinae with a range of photosynthetic pathways from C_3_ photosynthesis (*Oncidium cheirophorum*, *Oncidium maduroi*, *Rossioglossum krameri*, and *Oncidium sotoanum*) to weak CAM (*Oncidium panamense*, *Oncidium sphacelatum*, *Gomesa flexuosa* and *Rossioglossum insleayi*) and strong CAM (*Rossioglossum ampliatum*, *Trichocentrum nanum*, and *Trichocentrum carthagenense*). Phylogenetic analysis revealed the existence of two main *ppc* lineages in flowering plants, two main *ppc* lineages within the eudicots, and three *ppc* lineages within the Orchidaceae. Our results indicate that *ppc* gene family expansion within the Orchidaceae is likely to be the result of gene duplication events followed by adaptive sequence divergence. CAM-associated PEPC isoforms in the Orchidaceae probably evolved from several independent origins.

## Summary

Multiple isoforms of phospho*enol*pyruvate carboxylase genes were sequenced from related orchid species with distinct photosynthesic types. Phylogenetic analysis indicated that CAM-associated isoforms originated from gene duplications and adaptive sequence divergence.

## Introduction

Crassulacean acid metabolism (CAM) is one of three modes of photosynthesis found in vascular plants for the assimilation of atmospheric CO_2_. CAM differs from C_3_ and C_4_ photosynthesis in that CAM plants take up CO_2_ with low water expenditure at night when evaporative demand is low (Winter and Smith, 1996*b*; Cushman, 2001). CAM is phylogenetically widespread across 343 genera and 35 plant families comprising more than 6% of flowering plant species (Griffiths, 1989; Smith and Winter, 1996; Holtum *et al.*, 2007; Silvera *et al.*, 2010*a*). The multiple independent origins of CAM and the functional convergence of CAM traits in the many lineages in which it occurs suggest that the starting point for CAM evolution might have required relatively few genetic changes. However, this notion may be simplistic, given the complexity of the CAM pathway and the fact that several biochemical and anatomical requirements, and regulatory changes associated with gene expression patterns, are all tightly coordinated in CAM plants (Cushman *et al.*, 2008; Silvera *et al.*, 2010*a*). To gain insight into the evolutionary history of genes recruited for CAM function, the study of taxa containing many closely related species with contrasting photosynthetic pathways is experimentally helpful. In this context, we use tropical orchids as a study group, because CAM is widespread among epiphytes within this large family of vascular plants (Winter and Smith, 1996*a*; Silvera *et al.*, 2009, 2010a, *b*), and because species within the Orchidaceae exhibit a gradient of photosynthetic pathways ranging from C_3_ photosynthesis to weak- and strong-CAM modes (Silvera *et al.*, 2005, 2010*a*). Weak-CAM species show low-level CAM activity and typically obtain 5% or less of their carbon through the CAM pathway under well-watered conditions. Weak CAM appears to be common among neotropical orchid species (Silvera *et al.* 2005).

In vascular plants, phospho*enol*pyruvate carboxylase (PEPC; EC 4.1.1.31) belongs to a multigene family, and each member encodes an enzyme with specialized functions (Gehrig *et al*., 1995, 1998, 2001; Chollet *et al.*, 1996; Izui *et al.*, 2004; O’Leary *et al.*, 2011). In species performing C_4_ photosynthesis and CAM, one or more *ppc* genes encode isoforms of the enzyme that catalyse the fixation of atmospheric CO_2_ into C_4_-dicarboxylic acids. In CAM plants, CAM-specific isoforms catalyse the nocturnal, irreversible β-carboxylation of phospho*enol*pyruvate in the presence of HCO_3_^–^ and Mg^2+^ yielding oxaloacetate and inorganic phosphate. Oxaloacetate is then converted to malate, which is stored as malic acid in the vacuole. During the subsequent day, malic acid is decarboxylated, resulting in CO_2_ release and refixation by ribulose-1,5-bisphosphate carboxylase/oxygenase. This CO_2_-concentrating mechanism, or ‘CO_2_ pump’, suppresses photorespiration and improves water-use efficiency relative to that of C_3_ and C_4_ plant species (Cushman and Bohnert, 1997). Cytosolic and chloroplastic PEPC enzymes are found in photosynthetic organisms from higher plants and green algae to cyanobacteria and photosynthetic bacteria, and also in non-photosynthetic bacteria and protozoa (Chollet *et al.*, 1996; Izui *et al.*, 2004). In all plants, ‘housekeeping’ or non-photosynthetic isoforms of PEPC catalyse anapleurotic reactions to replenish biosynthetic precursors for the Krebs cycle. PEPC has many other physiological roles in plants that include maintaining cellular pH, supplying carbon to N_2_-fixing legume root nodules, absorbing and transporting cations in roots, control of stomatal movements, fruit maturation, and seed germination (Latzko and Kelly, 1983; Chollet *et al.*, 1996; Echevarria and Vidal, 2003; Izui *et al.*, 2004; O’Leary *et al.*, 2011; Singh *et al.*, 2012). While PEPC is mainly a cytosolic enzyme, a plastid-localized version of the enzyme, rice *Osppc4*, has been described and is involved in providing organic acids for ammonium assimilation in leaves (Masumoto *et al.*, 2010).

The currently available molecular data support the view that none of the C_4_ or CAM enzymes are unique to C_4_ or CAM plants (Westhoff and Gowik, 2004; Gowik and Westhoff, 2011), suggesting that these ubiquitous and functionally diverse isoforms served as starting points for the evolution of the C_4_ and CAM genes (Cushman and Bohnert, 1999; Monson, 1999). Furthermore, evidence from PEPC and comparative analysis of the C_4_-cycle enzymes in C_3_, C_3_–C_4_ intermediates, and C_4_ species in the genus *Flaveria* suggests that key amino acid residue changes are responsible for their acquisition of distinct kinetic and regulatory properties (Blasing *et al.*, 2000, 2002; Westhoff and Gowik, 2004). Similarly, phylogenetic analysis indicates that multiple origins of C_4_ photosynthesis in grasses and sedges are the likely result of recurring selection acting on a few amino acid positions of the PEPC enzyme within and across taxonomic scales (Christin *et al*., 2007, 2012*b*; Besnard *et al.*, 2009). Phylogenetic analyses also indicate that there was a single PEPC origin before the divergence of bacteria and plant lineages (Chollet *et al.*, 1996; Izui *et al.*, 2004; Westhoff and Gowik, 2004). CAM-specific PEPC isoforms are thought to have first evolved in response to water deficit from ancestral non-photosynthetic isoforms by gene duplication, followed by acquisition of transcriptional control sequences that mediate leaf- or photosynthetic-tissue-specific increases in mRNA expression (Gehrig *et al*., 2001, 2005; Taybi *et al.*, 2004). For example, seven distinct PEPC isoforms were recovered in the CAM species *Kalanchoe pinnata* (Lam.) Pers.: four isoforms from leaves and three from roots (Gehrig *et al.*, 2005). In *Mesembryanthemum crystallinum* L., a CAM-specific isoform was expressed during the induction of CAM, in addition to an uninduced housekeeping isoform (Cushman and Bohnert, 1999). Based on comparative studies of PEPC in many plant taxa including orchids performing CAM, Gehrig *et al.* (2001) predicted the clustering of PEPC isoforms according to their taxonomic position and specific function.

Previously, full-length *ppc* genes were characterized from bacteria, several vascular plant species, cyanobacteria, and protozoa (Izui *et al.*, 2004). The purpose of our study was to reconstruct the evolutionary history of PEPC in Orchidaceae. We have characterized the diversity of *ppc* genes in a phylogenetic context, using partial-length sequences from a closely related group of orchid species in the Oncidiinae that express photosynthetic pathways ranging from C_3_ photosynthesis to weak CAM and strong CAM. The results highlight the evolutionary diversification of *ppc* gene families and indicate the possible role of gene duplication and recruitment of *ppc* genes for CAM.

## Materials and methods

### Oncidiinae species and characterization

Eleven closely related species within the Oncidiinae with a range of photosynthetic pathways from C_3_ photosynthesis [*Oncidium cheirophorum* Rchb. f., *Oncidium maduroi* Dressler, *Oncidium sotoanum* R. Jimenez & Hagsater, and *Rossioglossum krameri* (Rchb. f.) M.W. Chase & N.H. Williams] to weak CAM [*Gomesa flexuosa* (G.Lodd.) M.W. Chase & N.H. Williams, *Oncidium panamense* Schltr., *Oncidium sphacelatum* Lindl., and *Rossioglossum insleayi* (Barker ex Lindl.) Garay & G.C. Kenn.] and strong CAM [*Rossioglossum ampliatum* (Lindl.) M.W. Chase & N.H. Williams, *Trichocentrum carthagenense* (Jacq.) M.W. Chase & N.H. Williams, and *Trichocentrum nanum* (Lindl.) M.W. Chase & N.H. Williams] were chosen as a study group for genetic studies of CAM based on carbon isotopic composition and titratable acidity measurements (Table 1; Silvera *et al.*, 2005). Oncidiinae represents one of the most highly diverse clades of orchids from the neotropics, with variation in chromosome number, vegetative features, photosynthetic mechanisms, and floral characteristics (Chase *et al.*, 2005). A phylogenetic reconstruction for these 11 species was performed using a matrix that included sequences of nuclear ribosomal DNA internal transcribed spacer 1 (*nrITS-1* and *-2*), plastid DNA regions *ycf1* (~1200bp portion from the 5′ end, and ~1500bp portion from 3′ end), *matK*, and the *trnH–psbA* intergenic spacer, as previously described (Neubig *et al.*, 2012) in each species, except for *O. maduroi*, which included only data for *nrITS-1* and *-2* and *ycf1*.

**Table 1.** Values of δ^13^C, leaf thickness, and titratable acidity for 11 species from the OncidiinaeTitratable acidity (∆H^+^) is represented as the difference between the mean±standard deviation of three replicates at morning and evening (Silvera *et al.*, 2005, and this study). Species are listed in order based on ∆H^+^ from C_3_ photosynthesis to weak CAM and strong CAM. FW, fresh weight; NS, not significant.

Species name	Leaf δ^13^C (‰)	Leaf thickness (mm)	H^+^ (evening) (µmol H^+^g^–1^ FW)	H^+^ (morning) (µmol H^+^g^–1^ FW)	∆H^+^	Photosynthetic pathway
*Oncidium sotoanum*	–25.2	0.25	2.9±0.4	2.7±1.6	–0.2 NS	C_3_
*Oncidium cheirophorum*	–27.4	0.36	29.9±3.3	30.2±2.7	0.3 NS	C_3_
*Rossioglossum krameri*	–31.7	0.35	14.4±2.5	14.8±2.4	0.4 NS	C_3_
*Oncidium maduroi*	–24.7	0.24	17.3±1.9	19.5±0.8	2.2 NS	C_3_
*Rossioglossum insleayi*	–22.5	1.10	16.3±0.5	34.9±12	18.60^*a*^	Weak CAM
*Oncidium panamense*	–26.2	0.54	11.5±0.7	33.2±0.3	21.7^*a*^	Weak CAM
*Oncidium sphacelatum*	–27.9	0.53	8.3±6.3	31.2±2.1	22.9^*a*^	Weak CAM
*Gomesa flexuosa*	–24.4	0.26	37.9±9.4	74.0±10.4	36.1^*a*^	Weak CAM
*Trichocentrum nanum*	–17.2	3.40	18.8±4.1	57.3±5.2	38.50^*a*^	Strong CAM
*Trichocentrum carthagenense*	–12.2	2.32	12.5±0.4	77.3±3.4	64.8^*a*^	Strong CAM
*Rossioglossum ampliatum*	– 15.3	1.59	5.5±1.3	153.5±3.6	148.0^*a*^	Strong CAM

^*a*^ Denotes significance between means of the morning and evening at *P*<0.05 as determined by Student’s *t*-test. NS, not significant.

### Gas-exchange measurements

Photosynthetic gas exchange was measured on attached, mature leaves of plants from each of the 11 orchid species targeted for this study. Measurements of 24h CO_2_ exchange demonstrate the proportions of CO_2_ fixed in the light and dark, respectively, and thus allow conclusions about the degree to which plants engage in CAM relative to C_3_ photosynthesis. Leaves were sealed inside a Plexiglass^®^ cuvette located within a controlled-environment chamber (Environmental Growth Chambers, Chagrin Falls, Ohio, USA). The hole in the cuvette through which the leaf was inserted was sealed with a non-porous synthetic rubber sealant (Terostat VII; Henkel-Teroson, Heidelberg, Germany). Prior to measurements, plants were well watered, fertilized once per week with a commercial 20:20:20 and/or 16:32:16 (N:P:K) fertilizer solution, and maintained in an open greenhouse. The diel temperature range within the greenhouse varied from a minimum of 20 °C to a maximum of 32.2 °C, and relative humidity varied from 80 to 100%. Daily light availability ranged from 5 to 70% of full sunlight and corresponded roughly to the natural light exposure of these species in the field. Plants were kept in their growth medium, which consisted of lightweight pumice aggregates and coarse synthetic sponge, to avoid dehydration before experiments. Due to the small size of *T. nanum* leaves, an entire plant of this species, including roots, was placed inside the cuvette. Net CO_2_ exchange was measured continuously using a flowthrough gas-exchange system (Walz, Effeltrich, Germany) operating at an airflow rate of 1.3 l min^–1^ and monitored with a LI-6252 infrared gas analyser (Li-Cor, Lincoln, NE, USA) operating in the absolute mode for up to 5 d. More than one individual per species was measured. The temperature inside the cuvette and chamber was 25 °C during the day and 22 °C during the night under an ambient CO_2_ concentration with a dew point of 18 °C and a light intensity of 300 µmol m^–2^ s^–1^ during the 12h light period.

### Titratable acidity determinations

Titratable acidity (∆H^+^) measurements were conducted as described previously and are presented as the difference between the mean±standard deviation of three replicate samples at dawn and dusk (Silvera *et al.*, 2005). Nocturnal acid accumulation reflects the magnitude of dark CO_2_ fixation but does not provide information on the uptake of atmospheric CO_2_ via C_3_ photosynthesis in the light.

### Plant material

Leaf samples for *ppc* gene analysis were obtained from mature leaves of mature orchids for 10 of the 11 species targeted for this study (*O. cheirophorum* was excluded from the gene analysis due to insufficient leaf material). Plants were grown in closed greenhouses at the University of Nevada, Reno, and the University of California, Riverside. Daily temperatures within the greenhouse varied from 17 to 35 °C, relative humidity varied from 40 to 80%, and the mean photon flux density was 200 µmol m^–2^ s^–1^. Plants were watered daily and nutrients were supplied once per week with a combination of slow-release fertilizer (Osmocote^®^ 19-6-12 formula; Scotts Company, Marysville, OH, USA) and commercial fertilizer solution (Schultz^®^ 19-31-17 formula; Spectrum Brands, Madison, WI, USA). Healthy leaves from each of 10 species were collected at 2 p.m. and 2 a.m. to account for putative circadian differences in the relative expression abundance of PEPC mRNAs. Root samples were also collected at the same times from mature plants and stored separately. All samples were flash frozen in liquid nitrogen immediately after harvesting, and stored at –80 °C until isolation of total RNA.

### RNA extraction

Total RNA was extracted using an RNeasy Midi kit (Qiagen, Valencia, CA, USA) with a modified polyethylene glycol RNA extraction method including high-molecular-weight polyethylene glycol (Gehrig *et al.*, 2000), which has proven successful for RNA isolation from succulent and non-succulent orchid tissues. RNA integrity was examined by 1% agarose gel electrophoresis, and RNA quality and concentration were examined using a NanoDrop ND-1000 UV-V spectrophotometer (NanoDrop Technologies, Rockland, DE, USA).

### Reverse transcription (RT)-PCR amplification and cloning

An 1100bp fragment was amplified by RT-PCR using two degenerate primers, as defined previously for PEPC (Taybi *et al.*, 2004; Gehrig *et al.*, 2005; Kore-eda *et al.*, 2005) and modified slightly for orchid specificity. The degenerate primers were: 5′-TCNGAYTCNGGVAARGAYGC-3′ (forward) and reverse 5′-GCDGCRATRCCYTTCATKG-3′ (reverse). Using a One-Step RT-PCR kit (Qiagen), 500ng of total RNA was reverse transcribed and amplified following the manufacturer’s instructions. Final concentrations of the reaction components were 400 µM for each dNTP, 1× Qiagen One-Step RT-PCR Buffer containing 12.5mM MgCl_2_, 2 µl of Qiagen One-Step RT-PCR Enzyme Mix, and 1 µM PEPC forward and reverse primers. The following temperature cycling conditions were used: reverse transcription at 50 °C for 30min, initial PCR activation at 95 °C for 15min, amplification for 39 cycles at 94 °C for 1min, 40 °C for 1min, and 72 °C for 1min, and final extension at 72 °C for 10min. RT-PCR was used to amplify a fragment of 1100bp encompassing the C-terminal third of the PEPC coding region. By using this partial sequence, distinct isoforms could be distinguished without the need to isolate the full 3000bp sequence, because this fragment was variable enough to allow differentiation among isoforms. The sequence also has a highly conserved active site that facilitates correct alignment and identification (Gehrig *et al.*, 2001; Izui *et al.*, 2004) (Supplementary Fig. S2 at *JXB* online). In this regard, the use of partial rather than full-length PEPC cDNA sequences has proven useful for molecular phylogenetic and taxonomic comparisons across species, thus saving time and financial resources for researchers interested in using PEPC as a molecular marker (Gehrig *et al.*, 2001). The RT-PCR products were purified by agarose gel electrophoresis, recovered using a QIAquick Gel Extraction kit (Qiagen), cloned into the TA-TOPO cloning vector pCR2.1 vector system (Invitrogen^TM^ Life Technologies, Carlsbad, CA, USA), and transformed into XL1-Blue or TOP10 competent *Escherichia coli* cells following the manufacturer’s instructions. Bacterial cells containing plasmids from 100–150 randomly selected clones per orchid species were grown in Terrific Broth liquid medium for 16h at 37 °C with vigorous shaking. The bacterial cells were then harvested by centrifugation at 13 000*g*, and the plasmids were purified using a Qiagen Plasmid Mini kit (Qiagen) following the manufacturer’s instructions. Selected cDNA clones were then analysed by *Eco*RI restriction enzyme digestion and electrophoresis on 1% agarose gels stained with 0.5 µg ml^–1^ of ethidium bromide. Selected plasmids were sequenced at the Nevada Genomics Center, University of Nevada, Reno, with a ABI BigDye™ Terminator Cycle Sequencing Ready Reaction kit, v3.1, and an ABI 3730 Sequence Analyzer (Applied Biosystems, Life Technologies, Grand Island, NY, USA), using the M13 forward (5′-TGTAAAACGACGGCCAGT-3′) and reverse (5′-GAGCGGATAACAATTTCACACAG-3′) primer sets. Over 1200 cDNA clones were sampled and sequenced from 10 of the 11 closely related Oncidiinae species targeted for this study, and 1000 cDNAs were selected and identified as PEPC isoforms (100 clones per species).

### PEPC sequence analysis

Raw sequences were edited manually by removing vector sequences using MacVector v.11.1 software (MacVector, Cary, NC, USA). Forward and reverse PEPC fragments were then assembled in MacVector. Over 1200 assembled sequences were verified by identifying conserved amino acid sequences using the Basic Local Alignment Search Tool (BLAST) within the non-redundant database at the National Center for Biotechnology Information, and were translated into the corresponding amino acid sequences. Multiple sequence alignments for both nucleotide and protein sequences were used to visually identify distinct PEPC isoforms within each species separately. Sequences that were identical within a species were considered the same isoform. PEPC isoforms present in each species were then named based on their relative abundance, so that *ppc1-o1* (letter ‘o’ for Oncidiinae) would correspond to the most abundantly transcribed isoform recovered by clone sampling in each species, followed by *ppc1-o2*, *ppc1-o3*, *ppc1-o4*, *ppc1-o5*, and *ppc1-o6*, respectively. By naming isoforms in this fashion, we could easily identify the isoforms that are abundantly expressed in leaves and roots when perfoming phylogenetic analysis. All isoforms were then renamed based on their position in the PEPC phylogenetic analysis (*ppc1-M1-o1*, *ppc1-M1-o2*, *ppc1-M2-o1*, *ppc-M2-o2*, and so forth).

### Rapid amplification of cDNA ends (RACE) amplification

The 3′ ends of the PEPC cDNA fragments for three species (*O. maduroi*, *O. panamense*, and *R. ampliatum*) were recovered using the 3′ RACE system (SMART^TM^ RACE cDNA Amplification, BD Bioscience Clontech, Mountain View, USA) following the manufacturer’s instructions and using gene-specific primers (GSPs) based on sequences obtained by the initial degenerate RT-PCR (Supplementary Table S1 at *JXB* online). RACE and larger amplified cDNA products were then sequenced using a BigDye™ Terminator Sequencing kit and an ABI 3730 Sequence Analyzer in the Nevada Genomics Center at the University of Nevada, Reno. Sequences were edited manually for base-call inaccuracies, and vector sequences were removed using MacVector v.11.1 software. Sequences were then assembled into the 1100bp isoform fragments for each species using the assembly project function in MacVector. To identify sequencing errors or chimaeras that were potentially formed during RACE, GSPs were also designed to confirm isoform identities (Supplementary Table S2 at *JXB* online). We only performed RACE amplification in three of the 10 species for which tissue was abundantly available.

### Phylogenetic analyses of *ppc*

Multiple sequence alignments that included 39 PEPC isoforms from the study species and 273 *ppc* sequences available in GenBank and Phytozome (Goodstein *et al.*, 2012) were obtained using the MUSCLE function (Edgar, 2004) in MEGA v.5.2.2 (Tamura *et al.*, 2011) and refined manually. The *ppc* sequences downloaded from GenBank included those from several orchid genera with CAM species, such as *Angraecum*, *Dendrobium*, *Epidendrum*, *Leptotes*, *Microcoelia*, *Phalaenopsis*, *Solenagis*, *Taeniophyllum*, and *Vanilla*. The *ppc* gene sequences from 63 plant genera and a sequence from the alga *Chara fragilis* Desv. (Supplementary Table S3 at *JXB* online) were also included in the alignment. Because phylogenetic trees based on *ppc* gene sequences from broad phylogenetic sampling have been shown consistently to produce two distinct lineages that are highly homologous but that diverged before the evolution of land plants (*ppc-1* and *ppc-2*), we focused our analysis only on sequences from *ppc-1*, which contain all of the CAM and C_4_-specific *ppc* genes (Gowik and Westhoff, 2011; Christin *et al.*, 2014). By using only *ppc-1* genes, we avoided ambiguities in the alignment files that could then be reflected in the phylogenetic analysis (Christin *et al.*, 2014). Phylogenetic trees were inferred with MrBayes v.3.2.1 (Ronquist and Huelsenbeck, 2003) following a general time-reversible model of nucleotide substitution with a γ-shaped parameter and a proportion of invariants (GTR+G+I). Two analyses were performed in parallel; each was composed of 16 chains, run for 20 000 000 generations, sampling a tree every 1000th generation after a burn-in period of 7 000 000 generations. A consensus tree was computed after the burn-in period. The convergence and appropriateness of the burn-in period were checked with Tracer (Rambaut and Drummond, 2007).

## Results

### Photosynthetic patterns and relationship of 11 Oncidiinae species

**Fig. 1.**
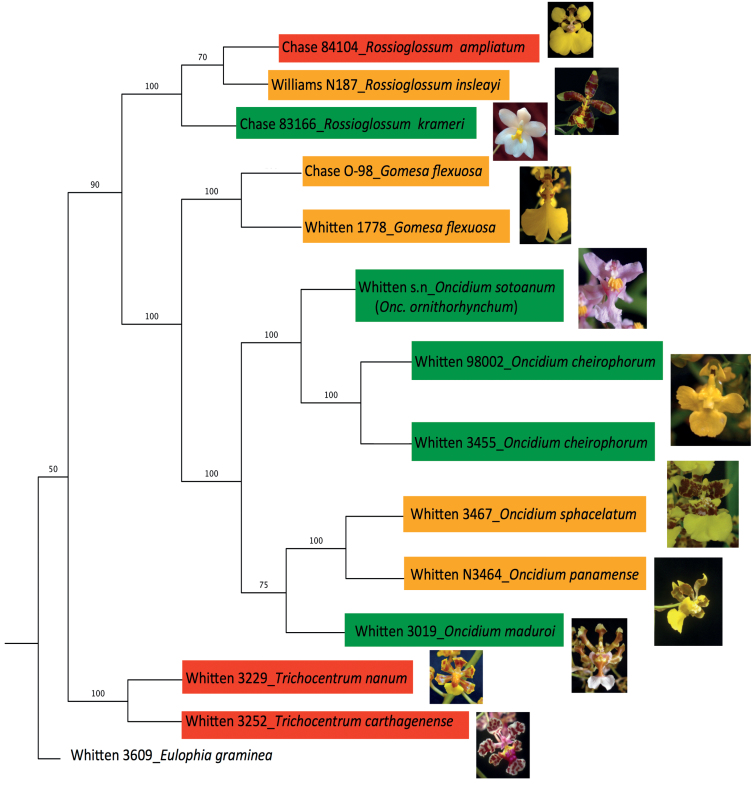
Phylogenetic relationship of 11 species from the Oncidiinae with photosynthetic pathways ranging from strong CAM (red shading) to weak CAM (yellow shading), and C_3_ photosynthesis (green shading). The phylogeny was reconstructed using sequences of *nrITS-1 and -2*, plastid DNA *ycf1*, *matk*, and the *trnH*–*psbA* intergeneric spacer. *Eulophia graminea* was used as the outgroup. Values on the branches represent bootstrap support (%). Accession numbers next to each species correspond to the voucher specimens deposited at the University of Florida Herbarium (FLAS). Representative images of corresponding floral morphology are shown to the right of each species designator. Photos by K. Silvera.

**Fig. 2.**
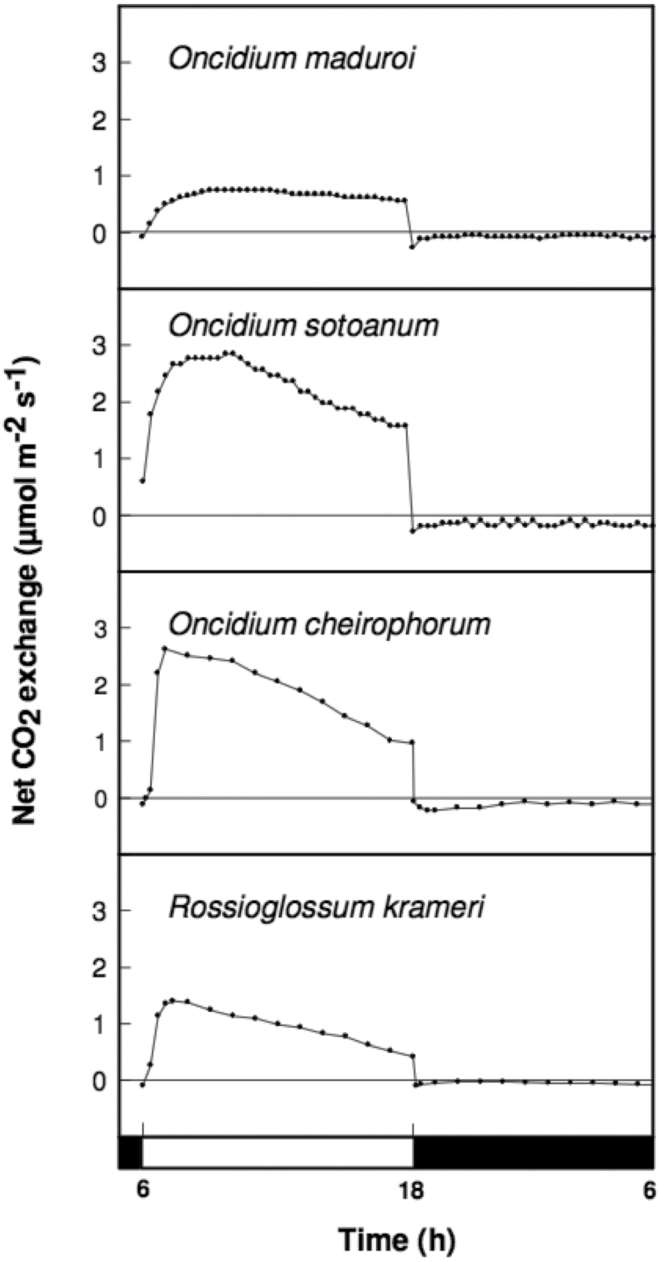
Continuous net CO_2_ exchange by C_3_ photosynthesis orchid species during a 12h light (open bar)/12h dark (filled bar) cycle period.

**Fig. 3.**
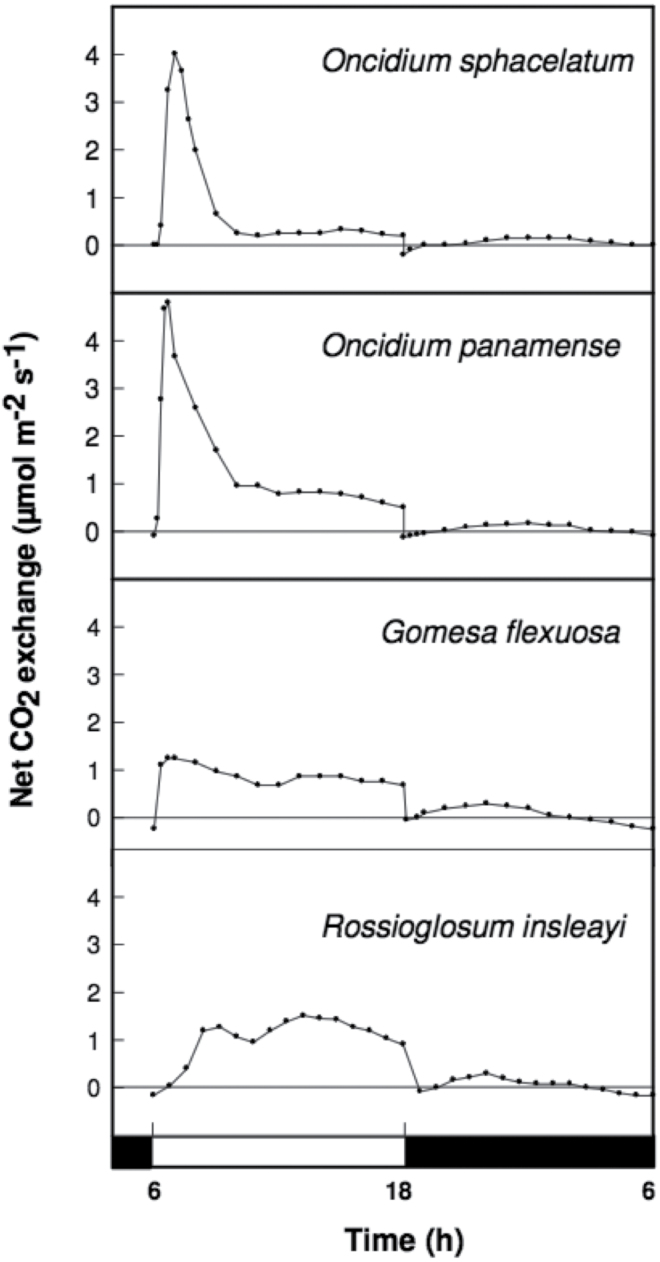
Continuous net CO_2_ exchange by weak-CAM orchid species during a 12h light (open bar)/12h dark (filled bar) cycle period.

**Fig. 4.**
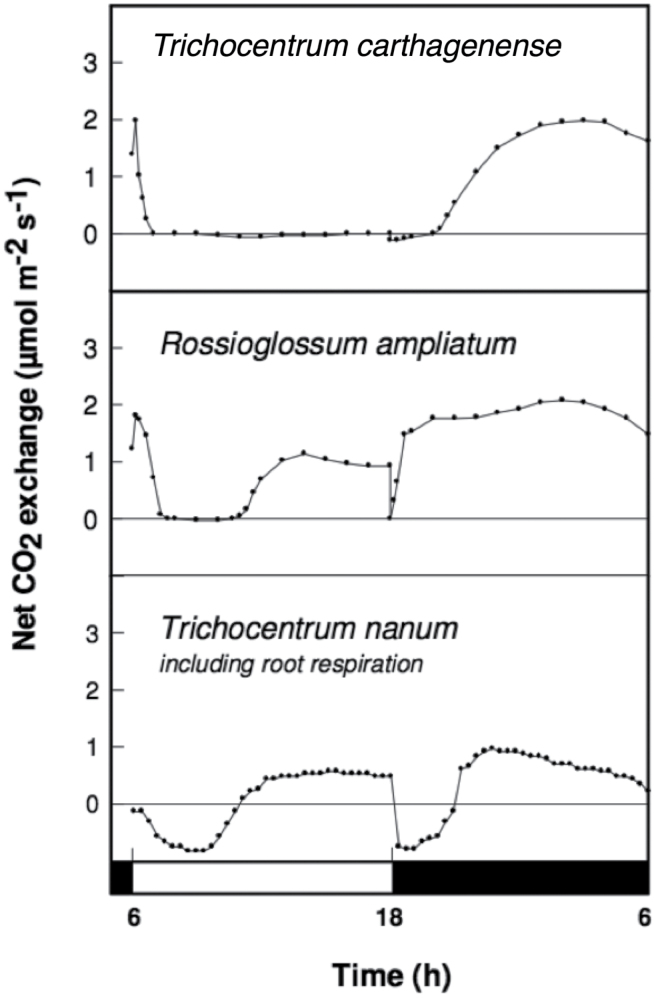
Continuous net CO_2_ exchange by strong-CAM orchid species during a 12h light (open bar)/12h dark (filled bar) cycle period.

Among the 11 Oncidiinae species used in this study, the genus *Rossioglossum* contained C_3_, weak-CAM, and strong-CAM species. *Oncidium* was represented by both weak-CAM and C_3_ species, whereas *Gomesa* was represented by one weak-CAM species and *Trichocentrum* contained two strong-CAM species (Fig. 1). The phylogenetic relationship of the 11 Oncidiinae species used in this study (Fig. 1) followed the nomenclature proposed by Neubig *et al.* (2012) and is consistent with the phylogenetic relationships within Oncidiinae *sensu* Chase inferred from 590 species (Neubig *et al.*, 2012). *O. maduroi*, *O. sotoanum*, *O. cheirophorum*, and *R. krameri* showed no nocturnal net CO_2_ uptake and therefore were considered C_3_ species (Fig. 2). *G. flexuosa*, *O. panamense*, *O. sphacelatum*, and *R. insleayi* clearly exhibited nocturnal net CO_2_ uptake, but its contribution to total carbon gain was small compared with CO_2_ uptake during the light period, so they were classified as weak-CAM species (Fig. 3). In these weak-CAM species, the majority of the CO_2_ was taken up during the day followed by a small amount of CO_2_ loss during the beginning of the night and limited net CO_2_ uptake throughout the night. *R. ampliatum*, *T. carthagenense*, and *T. nanum* were considered strong-CAM species based on their pronounced net CO_2_ uptake during the night (Fig. 4). *T. nanum* (a strong-CAM species; Fig. 4, bottom panel) showed daytime net CO_2_ uptake during the afternoon hours. However, the majority of its net CO_2_ uptake occurred during the middle of the night. Root respiration for *T. nanum* was included because the whole plant was measured within the cuvette due its small size. Compared with the other two strong-CAM species for which only leaf-gas exchange was determined, the presence of root respiration in the *T. nanum* experiment resulted in considerable CO_2_ loss during the early light and dark periods. Nonetheless, the strong-CAM character of the 24h CO_2_ gas-exchange pattern in this species was evident.

### Identification of multiple PEPC isoforms within Oncidiinae species

**Fig. 5.**
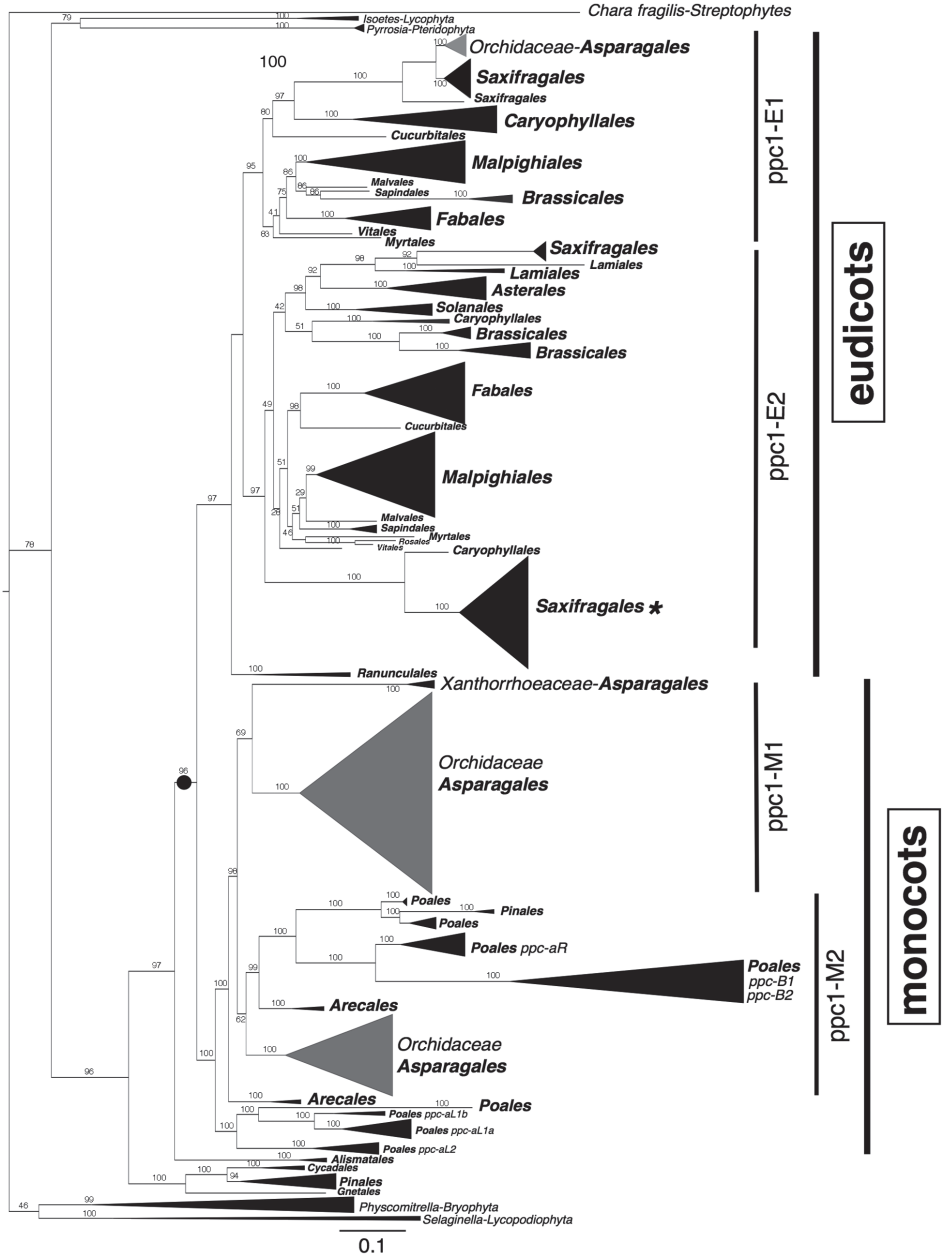
Phylogenetic relationship of 312 *ppc-1* gene sequences with emphasis on the Orchidaceae (shaded in grey). The phylogenetic tree was obtained through Bayesian analysis. Taxonomic groups are compressed based on plant orders, with the size of the triangle proportional to the number of sequences present in each clade. Orders are represented in bold, and the main *ppc* lineages are depicted on the right. Two main *ppc* lineages are highlighted in the tree (eudicots and monocots). The filled circle represents the duplication event before the split between eudicots and monocots. The asterisk represents a sequence of *Cycas revoluta* nested within a *Kalanchoe* clade (Saxifragales). Detailed information is available in Supplementary Fig. S1 at *JXB* online. Bar, expected substitutions per site.

Transcripts of between two and six isoforms of PEPC were sufficiently abundant to be recovered from each of the 10 species studied, based on our sampling strategy. Species performing C_3_ photosynthesis expressed two to three isoforms, whereas species with weak CAM expressed three to four isoforms, and species with strong CAM expressed four to six isoforms (Table 2). Multiple nucleotide sequence alignment of the 1100bp PEPC fragments from 39 isoforms recovered in this study and 273 sequences downloaded from GenBank and Phytozome showed 162 informative sites, 192 sites without gaps, and 186 variable sites. The *ppc-1* consensus tree generated from nucleotide sequence information from 312 species revealed two main lineages in flowering plants (eudicots and monocots; Fig. 5). The *ppc* sequences from the algal species *C. fragilis*, and those present in Bryophyta (*Physcomitrella*) and Lycopodiophyta (*Selaginella*), were sister to the *ppc* sequences from gymnosperms and angiosperms. The *ppc* lineage composed of *Pyrrosia*, a CAM fern (Fig. 5), and the aquatic CAM genus *Isoetes* was basal to the angiosperm and gymnosperm *ppc* lineages (Fig. 5).

**Table 2.** PEPC isoform counts from 10 species from Oncidiinae based on relative abundance of clone samplingFour additional isoforms were recovered using GSP by RACE amplification.

Species name	Isogene	Relative abundance (%)	Functional designation
*Oncidium maduroi* (C_3_)	*ppc1-M1-o1*	88	C_3_
	*ppc1-M2-o2*	11	C_3_
	*ppc1-M1-o3*	1	C_3_
*Rossioglossum krameri* (C_3_)	*ppc1-M1-o1*	92	C_3_
	*ppc1-M2-o2*	8	C_3_
*Oncidium sotoanum* (C_3_)	*ppc1-M1-o1*	86	C_3_
	*ppc1-M2-o2*	10	C_3_
	*ppc1-M1-o3*	4	C_3_
*Gomesa flexuosa* (weak CAM)	*ppc1-M1-o1*	66	CAM
	*ppc1-M1-o2*	31	C_3_
	*ppc1-M1-o3*	2	C_3_
	*ppc1-M2-o4*	1	C_3_
*Oncidium sphacelatum* (weak CAM)	*ppc1-M1-o1*	96	CAM
	*ppc1-M1-o2*	2.5	C_3_
	*ppc1-M1-o3*	1	C_3_
*Oncidium panamense* (weak CAM)	*ppc1-M1-o1*	73	CAM
	*ppc1-M1-o2*	19	C_3_
	*ppc1-M2-o3*	7.5	C_3_
	*ppc1-M1-o4*	by RACE	C_3_
*Rossioglossum insleayi* (weak CAM)	*ppc1-M1-o1*	76	CAM
	*ppc1-M1-o2*	11	C_3_
	*ppc1-M2-o3*	10	C_3_
	*ppc1-M1-o4*	2	C_3_
*Rossioglossum ampliatum* (CAM)	*ppc1-M1-o1*	70	CAM
	*ppc1-M1-o2*	20	C_3_ /CAM
	*ppc1-M2-o3*	5	C_3_
	*ppc1-M2-o4*	5	C_3_
	*ppc1-M1-o5*	by RACE	C_3_
	*ppc1-M2-o6*	by RACE	C_3_
*Trichocentrum carthagenense* (CAM)	*ppc1-M1-o1*	84	CAM
	*ppc1-M1-o2*	9	C_3_ /CAM
	*ppc1-M2-o3*	4	C_3_
	*ppc1-M1-o4*	3	C_3_
	*ppc1-M1-o5*	by RACE	C_3_
*Trichocentrum nanum* (CAM)	*ppc1-M1-o1*	59	CAM
	*ppc1-M2-o2*	20	C_3_ /CAM
	*ppc1-M1-o3*	16	C_3_
	*ppc1-M2-o4*	4	C_3_
	*ppc1-M1-o5*	1	C_3_

**Fig. 6.**
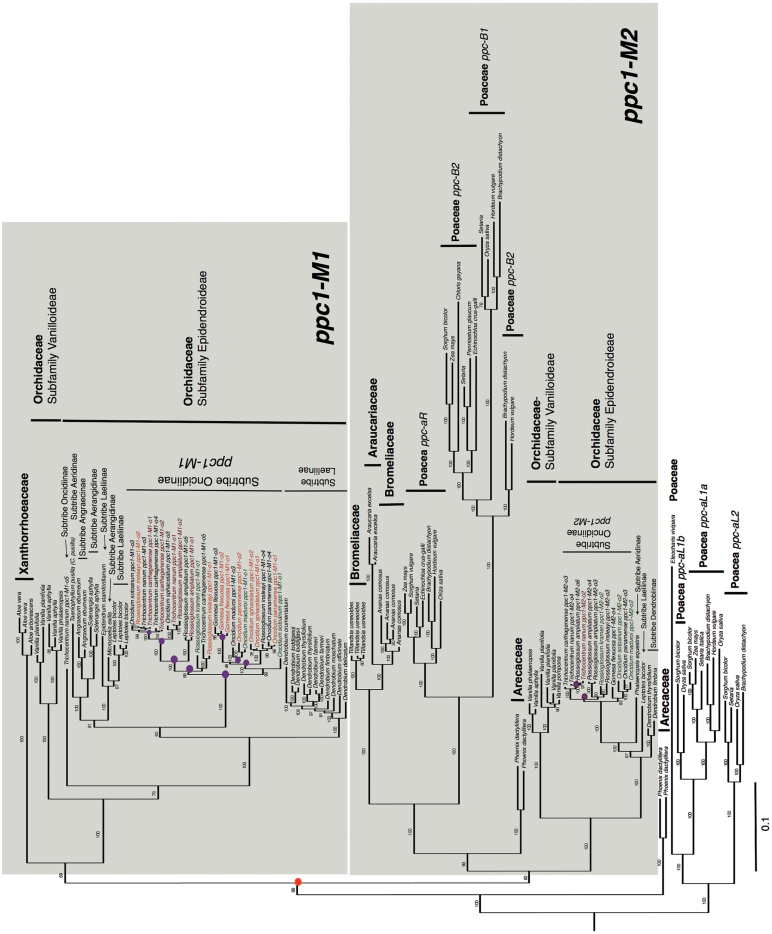
Phylogenetic relationship of monocot *ppc1* sequences with emphasis on the Orchidaceae. The phylogenetic tree was obtained through Bayesian analysis. Plant families are represented in bold. Two lineages within monocots are highlighted in grey (*ppc1-M1* and *ppc1-M2*). Orchid subfamilies and subtribes are depicted on the right. The two most abundantly transcribed *ppc* isoforms for C_3_, weak-CAM, and strong-CAM orchid species are highlighted in green, orange, and red, respectively. The red filled circle represents an early duplication event leading to lineages *ppc1-M1* and *ppc1-M2*. Magenta filled circles represent duplication events within Orchidaceae lineages. Detailed information is available in Supplementary Fig. S1. Bar, expected substitutions per site.

The eudicot lineage was monophyletic and composed of two well-supported clades (*ppc1-E1* and *ppc1-E2*; Fig. 5 and Supplementary Fig. S1). The position of these clades was consistent with those described by Christin and Besnard (2009) and Christin *et al.* (2014). Gene duplication events that led to the six gene lineages of *ppc* in C_4_ grasses are shown within the monocot lineages (namely *ppc-aL1a*, *ppc-aL1b*, *ppc-aL2*, *ppc-aR*, *ppc-B1*, and *ppc-B2*, Figs 5 and 6) and follow the nomenclature proposed by Christin and Besnard (2009). The monocot lineage in the current study appeared to be paraphyletic because some monocot Orchidaceae *ppc* sequences were embedded within the eudicots *ppc1-E1* lineage (Fig. 5, represented by the grey clade).

Gene duplication events led to three *ppc* lineages in the Orchidaceae: two well-supported lineages within the monocot lineage (*ppc1-M1* and *ppc1-M2*; Fig. 6), and a third lineage, which is embedded within the eudicot *ppc1-E1* lineage (Fig. 5). The monocot lineage contained all of the 39 transcribed isoforms recovered in this study (*ppc1-M1* and *ppc1-M2*; Fig. 6). The most abundantly transcribed isoform of all the Oncidiinae species recovered in this study grouped within the *ppc1-M1* lineage (Fig. 6, *ppc1-M1-o1* represented in red, orange, and green). The most abundantly transcribed isoforms for weak-CAM species (e.g. *G. flexuosa ppc1-M1-o1* and *ppc1-M1-o2*, *O. sphacelatum ppc1-M1-o1* and *ppc1-M1-o2*, *O. panamense ppc1-M1-o1* and *ppc1-M1-o2*, and *R. insleayi ppc1-M1-o1* and *ppc1-M1-o2*, represented in orange in Fig. 6) clustered together with the most abundantly transcribed isoforms for strong-CAM species (e.g. *R. ampliatum ppc1-M1-o1* and *ppc1-M1-o2*, *T. carthagenense ppc1-M1-o1* and *ppc1-M1-o2*, and *T. nanum ppc1-M1-o1*, represented in red in Fig. 6). The most abundantly transcribed isoform for C_3_ species (e.g. *O. maduroi ppc1-M1-o1*, *R. krameri ppc1-M1-o1*, and *O. sotoanum ppc1-M1-o1*, represented in green in Fig. 6) also clustered with the most abundantly transcribed isoforms for weak- and strong-CAM species. The phylogenetic similarity between the two most abundantly transcribed isoforms for weak- and strong-CAM species (*ppc1-M1-o1* and *ppc-M1-o2*) and the most abundantly transcribed isoform for C_3_ species (*ppc1-M1-o1*) suggested that these orthologous isoforms have similar expression patterns and abundance and are probably involved in the same functions. The *ppc* sequences from Oncidiinae genera such as *Gomesa*, *Oncidium*, *Rossioglossum*, and *Trichocentrum* tended to separate into two distinct groups (*ppc1-M1* and *ppc1-M2*), and this pattern was also consistent within orchid genera in which several isoforms were reported such as *Vanilla*, *Leptotes*, and *Dendrobium* (Fig. 6). Within the monocot lineage, Orchidaceae *ppc* sequences followed the species relationships in both *M1* and *M2* clusters. For example, *ppc1-M1* was composed of *ppc* sequences from closely related Oncidiinae species (species within *Gomesa*, *Oncidium*, *Rosioglossum*, and *Trichocentrum*; Fig. 6), and *ppc1-M1* was sister to *ppc* sequences from other species in the subfamily Epidendroideae (species within subtribes Laeliinae, Aeridinae, Angraecinae, Aerangidinae; Fig. 6). The *ppc* sequences from the subfamily Epidendroideae were sister to *ppc* sequences from *Vanilla* (subfamily Vanilloideae) and *T. nanum ppc1-M1-o5* (Fig. 6). A gene duplication event probably occurred before the diversification of Oncidiinae *ppc1-M1* and *ppc1-M2* (Fig. 6, represented by a red filled circle). At least 10 gene duplication events were apparent within Oncidiinae *ppc* lineages and were related to *ppc* gene expansion in weak-CAM and strong-CAM species (Fig. 6, represented by magenta filled circles in *ppc1-M1* and *ppc1-M2*). Eight gene duplication events occurred within Oncidiinae *ppc1-M1*, four of which were linked to gene family expansion in lineages leading to the weak-CAM species *O. sphacelatum*, *O*. *panamense*, and *R. insleayi*, and four were linked to gene family expansion in lineages leading to the strong-CAM species *R. ampliatum*, *T. carthagenense*, and *T. nanum* (Fig. 6). Within Orchidaceae *ppc1-M2*, two gene duplication events were linked to gene family expansion, one of which occurred in a lineage leading to *R. ampliatum*, and in another leading to *T. nanum* (Fig. 6, represented by filled circles in *ppc1-M2*).

## Discussion

The Oncidiinae is one of the most diverse clades within the Orchidaceae, with a wide range of contrasting characteristics such as vegetative morphology, floral variation, chromosome number, and pollination systems (Neubig *et al.*, 2012). Our results indicate that Oncidiinae also shows contrasting photosynthetic types, as demonstrated by gas exchange, titratable acidity, leaf thickness, and isotopic composition measurements (Table 1, Figs 2–4). Most species within the Oncidiinae are epiphytes in habitats with intermittent water availability, and many exhibit CAM photosynthesis (Silvera *et al.*, 2009). The degree of CAM expression in orchid species correlates with leaf thickness or succulence (Winter *et al.*, 1983), reduced intercellular air spaces, and large mesophyll cell size (Nelson *et al.*, 2005), with a minimum necessary cell volume per unit leaf area for nocturnal acid storage. Therefore, thin-leaved, weak-CAM species are predicted to exhibit a limited degree of nocturnal net CO_2_ uptake (Fig. 3) when compared with thick-leaved, strong-CAM species (Fig. 4). Indeed, weak-CAM species in the Oncidiinae showed patterns of nocturnal CO_2_ uptake typical of CAM species but at a greatly reduced magnitude. Although their δ^13^C values were within the C_3_ range, these species exhibited statistically significant differences between evening and morning titratable acidity, which is indicative of weak CAM. Species with δ^ 13^C values characteristic of C_3_ species can obtain up to one-third of their carbon via the CAM pathway (Winter and Holtum, 2002). In contrast, gas-exchange measurements for the thin-leaved C_3_ photosynthetic species *O. maduroi*, *O. sotoanum*, *O. cheirophorum*, and *R. krameri* showed net CO_2_ uptake exclusively during the daytime and a slight CO_2_ loss during the night (Fig. 2). Strong-CAM species with thick leaves such as *R. ampliatum*, *T. carthagenense*, and *T. nanum* exhibited most of their carbon gain at night (Fig. 4).

Differences in PEPC isoform numbers and relative mRNA abundance using 10 closely related Oncidiinae species were associated with the capacity to perform CAM, as measured by 24h net CO_2_ gas exchange. Based on our RT-PCR approach, as many as six isoforms were observed in weak- and strong-CAM species, and one to two putative CAM-specific PEPC isoforms were identified based on their relative abundance (Table 2) and position in the phylogenetic tree (Fig. 6, orthologous *ppc1-M1-o1* and *ppc-M1-o2* sequences represented in red and orange). The increase in number of PEPC isoforms associated with weak- and strong-CAM species could be the result of a single gene duplication event, gene duplication due to polyploidy, or different alleles of the same gene. Oncidiinae CAM species have a significantly higher DNA content compared with weak-CAM and C_3_ species (J.C. Cushman, unpublished data). A possible explanation for the expansion of the *ppc* gene family is that repeated genome duplication events leading to polyploidy in orchids have created an increased pool of duplicated genes and alleles suitable for CAM. Therefore, the presence of multiple genomes might confer an advantage for adaptive evolution (Hegarty and Hiscock, 2007). Genomic information is needed to verify the number of isoforms present in each species and the subsequent orthology or paralogy of the *ppc* sequences found in this study, and to identify missing gene lineages that could not be recovered with our PCR-based sampling approach. Even so, the present study suggests that the *ppc* gene family in Oncidiinae orchids has probably undergone gene family expansion during the evolutionary establishment of weak and strong CAM as evidenced by at least eight gene duplication events revealed by the phylogenetic tree analysis.

The identification of two distinct *ppc* clades in flowering plants indicates that these two lineages evolved independently of each other, and that this event occurred early in the diversification of *ppc* lineages (Christin and Besnard, 2009; Christin *et al.*, 2012*b*). Within the Oncidiinae, a gene duplication event occurred early in the diversification of *ppc1-M1* and *ppc1-M2* lineages. Several *ppc1-M1* genes, presumably derived from gene duplication events (Fig. 6, represented by filled circles), evolved a role in CAM photosynthesis (Fig. 6, represented by red and orange sequences), whereas genes within *ppc1-M2* maintained anapleurotic roles. This phylogenetic grouping of PEPC isoforms into distinct sister clades (*ppc1*-*M1* and *ppc1*-*M2*) was also evident for other subtribes within the subfamily Epidendroideae (e.g. subtribe Laeliinae, subtribe Aeridinae, and subtribe Dendrobiinae; Fig. 6) and for the subfamily Vanilloideae PEPC isoforms.

Distinct Oncidiinae PEPC isoforms associated exclusively with root tissue were not identified in the current study. Gehrig *et al.* (2005) found three root isoforms of PEPC, each of which contained an insertion of 8 aa towards the C-terminal end of the enzyme in *K. pinnata*, a strong-CAM species. Putative root PEPC isoforms from the orchid species studied here showed isoforms that were identical to those found in leaves, and no insertions were evident. Because aerial roots in epiphytic orchids can engage in C_3_ photosynthesis (except for leafless orchids in which roots perform CAM; Winter *et al.*, 1985), the *ppc* genes found in roots of epiphytic orchids are most likely the same as those found in leaves (Kwok-ki *et al.*, 1983). Interestingly, *T. nanum ppc1-M1-o5* (Fig. 6) was quite distinct from all other isoforms in the Oncidiinae *ppc* sequences, and was positioned close to *Vanilla*, a distantly related orchid group (Fig. 6). This very-low-abundance isoform might be difficult to recover, which might explain why it was not previously reported in closely related species. The many low-abundance isoforms (*ppc1-o3* to *ppc1-o6*) found in this study might be the result of functional diversification of paralogous genes involved in non-photosynthetic PEPC functions (i.e. housekeeping or anapleurotic functions). The finding of a third *ppc* Orchidaceae lineage composed of three genera (*Microcoelis*, *Leptotes*, and *Solenangis*) outside the monocots and nested within the eudicots, and a *Cycas revoluta ppc* sequence nested within *Kalanchoe* sequences, is puzzling. Further investigation is needed to rule out the possibility that these sequences are the result of contamination or horizontal gene transfer. Additional quantitative and temporal expression analysis of mRNAs for all isoforms in this study is needed to confirm their putative functional contributions to CAM. Similarly, more genomic and transcriptomic data from orchid species are needed to confirm and identify *ppc* lineages in orchid genomes, and to recover isoforms that were missed due to potentially incomplete PCR-based sampling.

The PEPC isoforms most abundantly transcribed in C_3_ Oncidiinae species clustered closely with those of strong-CAM and weak-CAM species (Fig. 6, Oncidiinae species sequences highlighted in colors, *ppc1-M1*), suggesting that there is no specific convergence of amino acid changes or selective pressure towards amino acid changes linked to CAM function, as there is in C_4_ species (Christin *et al.*, 2012*b*). The most abundantly transcribed isoforms in cDNAs isolated from leaf and root photosynthetic tissues of C_3_, weak-CAM, and strong-CAM Oncidiinae species were orthologous, suggesting that these isoforms might be involved in similar functions and that they have a role in nocturnal CO_2_ uptake in species with weakly and strongly expressed CAM. All of the other Orchidaceae *ppc* sequences available from GenBank and used in this study belonged to strong-CAM species. There were no *ppc* sequences available from C_3_ orchid relatives, making it impossible to test whether *ppc* sequences from potentially closely related C_3_ species within the subfamilies Epidendroideae and Vanilloideae would have clustered in the same manner as those from strong-CAM species. This deficiency highlights the utility of conducting gene family surveys with closely related species with contrasting photosynthetic pathways to elucidate the molecular genetic underpinnings of photosynthetic pathway evolution.

Several scenarios could explain the diversification of PEPC isoforms. In one scenario, a *ppc* gene duplication event occurred early in the diversification of plants producing two clades: one clade with several duplicated sequences, one of which underwent recruitment for CAM through neofunctionalization, while the other clade contained sequences that retained the ancestral function. In general, neofunctionalizations require changes in gene expression (Bräutigam *et al.*, 2011; Gowik and Westhoff, 2011) and/or amino acid substitutions to confer an entirely new function (Zhang, 2003). A second scenario involves a change in regulation. In this scenario, a gene duplication event from a *ppc* ancestral gene with dual functionality underwent regulatory changes that determined whether it would perform in CAM or C_3_ photosynthesis. This implies that neofunctionalization does not need to be linked to changes in amino acid positions, and that C_3_ paralogous genes can be recruited for CAM function through subfunctionalization, in which one of the duplicated genes becomes better at performing one of the functions of the progenitor genes (Hughes, 1999; Zhang, 2003). Perhaps the most likely scenario in the evolution of PEPC in orchids is the former, as suggested by the close clustering of *ppc* sequences from C_3_, weak-CAM, and strong-CAM species that exhibited greater transcript abundance (Fig. 6, orthologous *ppc1-M1* genes, represented in red, orange, and green) relative to the other gene family members. This scenario suggests that increased transcript abundance occurs prior to the transition to the CAM phenotype. Also, the recurrent independent origin of CAM in distantly related plant clades (Keeley and Rundel, 2003), provides evidence that the evolution of CAM probably involves the use and modification of genes that are already present in the C_3_ ancestors of these species. Alternatively, cryptic genetic variants present in common ancestral populations could come to be expressed, and subsequently increase in frequency, in multiple descendent lineages under similar selection regimes (Barrett and Schluter, 2007; West-Eberhard *et al.*, 2011). This hypothesis envisions the presence of unexpressed PEPC alleles suitable for CAM and/or C_4_ photosynthesis in the genome of ancestral distantly related C_3_ species for millions of years, which can then be incorporated into CAM or C_4_ species through increased expression of either formerly cryptic or universally adaptive genes. These alleles could then become fixed through natural selection in populations in which they are suited to a new photosynthetic mode (West-Eberhard *et al.*, 2011; Christin *et al.*, 2012*a*). There is increasing evidence for the importance of cryptic and standing genetic variation in evolution (Gibson and Dworkin, 2004; Barrett and Schluter, 2007; McGuigan and Sgro, 2009) and no special mechanism is required for such variation to explain the convergent use of ancestral alleles in a new context.

Within the Orchidaceae, the presence of CAM is evolutionarily labile and prone to parallel evolution and reversal events especially within clades that contain large numbers of epiphytic species (subfamily Epidendroideae; Silvera *et al.*, 2009, 2010*a*). The association of CAM with semi-arid or arid environments and microhabitats, or other stressful conditions, suggests a role for environmental influences in its recurrent origin and evolution (West-Eberhard, 2003). This suggestion is also supported by the observation that the extent of CAM expression often correlates with the degree of specialized adaptations to more xeric ecological niches (Kluge *et al.*, 2001; Pierce *et al.*, 2002; Zotz, 2004). The recurrent evolution of CAM reflects strong selection under conditions in which CAM might afford an advantage, as in the epiphytic habitat of orchids. Advantageous biochemical shifts and molecular genetic rearrangements could modulate changes in mRNA or protein expression patterns from C_3_ to CAM, and imply a direct role for environmental cues in allowing selection to act on variation in underlying genotypes. Also, structural precursors such as enlarged vacuoles and tight cell packing may need to be present for the evolutionary progression from C_3_ to CAM to occur (Sage, 2002). After CAM becomes established within a lineage, the expression of CAM can be plastic or environmentally sensitive, as illustrated by the existence of ‘facultative’, ‘inducible’, or ‘optional’ CAM species that engage in CAM in response to environmental stimuli such as water-deficit stress (Winter, 1985; Griffiths, 1988; Winter *et al.*, 2008; Winter and Holtum, 2014). This hypothesis is also supported by the observation that the weak-CAM species contain increased numbers of gene duplication events compared with the C_3_ species, and that these species have added the novel capacity for net dark CO_2_ fixation to their continued capacity to express mostly C_3_ photosynthesis. This flexibility, combined with the ubiquity of enzymes required to perform CAM, might explain why multiple independent origins of CAM, as well as reversals, have been observed within the Orchidaceae (Silvera *et al.*, 2009).

In summary, several lines of evidence presented here suggest that the evolution of CAM *ppc* genes in orchids involved gene duplication coupled with the recruitment of specific gene family member for photosynthetic pathway-specific functions. First, using our cloning approach, increased numbers of expressed PEPC isoforms were detected as sampling proceeded from C_3_ photosynthesis to weak-CAM and strong-CAM species within closely related species of the Oncidiinae. Secondly, phylogenetic analysis revealed that *ppc* genes with the greatest relative transcript abundance from C_3_, weak-CAM, and strong-CAM species grouped together. This observation suggests that the identified increases in PEPC mRNA expression typical of CAM-specific isogenes were acquired before the species diverged and might indicate parallel rather than convergent evolutionary tracks for these specific gene lineages.

The current Oncidiinae *ppc* dataset lays a strong foundation for future comparisons of gene lineages expressed in different tissues of C_3_, weak-CAM, and strong-CAM species. However, more comprehensive transcriptomic and genomic datasets based on deep-sequencing methods are required to identify potentially missing gene lineages and possible new PEPC isoforms. For example, gene family members with low-abundance transcripts identified in the current study might exhibit higher transcript abundance in other species, leading to a more detailed understanding of lineage-specific gene recruitment patterns among diverse CAM species. In addition to conducting detailed sampling of all possible tissues types, including leaf, pseudobulb, and root tissues, future works should include the sampling of these tissues over more detailed time courses in order to identify gene family members and lineages that have acquired pronounced diel or circadian mRNA expression patterns, which are likely to be useful diagnostic indicators for gene recruitment to CAM-specific function (Silvera *et al.*, 2010*a*). In conclusion, this study provides clear evidence for the roles of gene duplication and neofunctionalization within *ppc* gene lineages in the evolutionary progression of CAM within the Oncidiinae.

## Supplementary data

Supplementary data is available at *JXB* online.

Supplementary Table S1. Gene-specific primer sets used for 3′ RACE amplification of the cDNA fragment coding for PEPC isoform for three species from Oncidiinae.

Supplementary Table S2. Gene-specific primer sets used for confirmation of the cDNA fragment recovered by RACE coding for PEPC isoform for three species from Oncidiinae.

Supplementary Table S3. The *ppc* sequence names used for phylogenetic analyses.

Supplementary Fig. S1. Phylogenetic tree of 312 *ppc-1* gene sequences.

Supplementary Fig. S2. Protein sequence alignment for 28 PEPC isoforms and 11 PEPC isoforms recovered by RACE for 10 Oncidiinae species.

Supplementary Data

## Abbreviations:

∆H^+^: titratable acidity
CAM: crassulacean acid metabolism
GSP: gene-specific primer
PEPC: phospho*enol*pyruvate carboxylase
RACE: rapid amplification of cDNA ends
RT-PCR: reverse transcription-PCR.
