# Supplementary material for: Multiple isoforms of phosphoenolpyruvate carboxylase in the Orchidaceae (subtribe Oncidiinae): implications for the evolution of crassulacean acid metabolism
Source: J Exp Bot. 2014 Jun 9;65(13):3623–36. doi: 10.1093/jxb/eru234 (PMC4085970; doi:10.1093/jxb/eru234)
Supplement: Supplementary Data [file supp_65_13_3623__index.html]

Multiple isoforms of phosphoenolpyruvate carboxylase in the Orchidaceae (subtribe Oncidiinae): implications for the evolution of crassulacean acid metabolism — Multiple isoforms of phosphoenolpyruvate carboxylase in the Orchidaceae (subtribe Oncidiinae): implications for the evolution of crassulacean acid metabolism — Supplementary Data 

# Multiple isoforms of phospho*enol*pyruvate carboxylase in the Orchidaceae (subtribe Oncidiinae): implications for the evolution of crassulacean acid metabolism

## Supplementary Data

Data files

**Files in this Data Supplement:**

- Supplementary Data - Supplementary Data
- Supplementary Data - Supplementary Data
