# Supplementary material for: Multiple isoforms of phosphoenolpyruvate carboxylase in the Orchidaceae (subtribe Oncidiinae): implications for the evolution of crassulacean acid metabolism
Source: J Exp Bot. 2014 Jun 9;65(13):3623–36. doi: 10.1093/jxb/eru234 (PMC4085970; doi:10.1093/jxb/eru234)
Supplement: Supplementary Data [file supp_eru234_jexbot117085_file001.pdf]

**Title: Multiple isoforms of phospho*enol*pyruvate carboxylase in the Orchidaceae (subtribe Oncidiinae): implications for the evolution of crassulacean acid metabolism**

**Authors:** Katia Silvera[*†], Klaus Winter[*], B. Leticia Rodriguez[†], Rebecca L. Albion[†], and John C. Cushman[†]

**Table S1.** Gene specific primer sets (GSP 5'– 3' position) used for 3'RACE amplification of the cDNA fragment coding for *Ppc* isoform for three species from the subtribe Oncidiinae. GSP1-1 to GSP1-23 were used for the first round of amplification, and GSP2-1 to GSP2-23 were used in the second round of amplification to reduce non-specific products

| | |
|---|---|
| GSP1-1 | AACTCTGAACGTCTGCCAAGCCTACGCG |
| GSP1-2 | AACTCTGAACGTTTGCCAAGCCTACACA |
| GSP1-3 | GACCCTGAATGTGTGCCAAGCCTTCACC |
| GSP1-4 | GACGCTGAATGTGTGCCAAGCCTTCACC |
| GSP1-5 | AACTCTGAACGTTTGCCAAGCCTACACG |
| GSP1-6 | TACTCTGAACGTTTGCCAAGCCTGCACA |
| GSP1-7 | GACCCTGAATGTGTGCCAAGCCTTCACT |
| GSP1-8 | GACCCTGAATGTGTGCCAAGCTTTCACC |
| GSP1-21 | CGACTCAGGCTCCGCTACCCG |
| GSP1-22 | TTGGCAGTTGTACAAGGCTCAGGAG |
| GSP1-23 | GGCAGCTATATAAGACTCAAGG |
| GSP2-1 | GCGAATCAGAGATCCAAGCGATCATGG |
| GSP2-2 | GCGGATCCGAGATCCAAGCAATGGTCA |
| GSP2-3 | GCGAATCAGAGATCCAAGCGATCATCG |
| GSP2-4 | GCGAATCAGAGATCCGAGCGATCATCA |
| GSP2-5 | GCGAATCAGAGATCCGAGCGATCATCC |
| GSP2-6 | GCGAATCAGAGATCCAAGCGATCATGG |
| GSP2-7 | AAGGATCAGAGACCCAAATTTTCTTGT |
| GSP2-8 | AAGGATCAGAGACCCAAAATTTCTTGT |
| GSP2-9 | AAGGATCAGAGATCCAAATTTTCTTGT |
| GSP2-10 | AAGAATCAGAGACCCAAATTTTCTTGTG |
| GSP2-12 | GCGGATCAGAGATCCAAGCGATCGTCA |
| GSP2-13 | GCGGATCAGAGATCCAAGCGATCATCG |
| GSP2-14 | GCGGATCAGAGATCCAAGCAATGGTCA |
| GSP2-15 | GCGGATCCGAGATCCAAGCGACGGTCA |
| GSP2-16 | AAGGATCAGAGACCCAAATTTTCATGT |
| GSP2-21 | AACGTTTGCCAAGCCTACACGC |
| GSP2-22 | GGCTCAGGAGGATCTCATAAAAGTG |
| GSP2-23 | GAAAGTAGCCAAGGAATTTGGA |

**Table S2.** Gene specific primer sets (5'– 3' position) used for confirmation of the cDNA fragment recovered by RACE coding for *Ppc* isoform for three species from the subtribe Oncidiinae species. *Oncidium maduroi* (MAD), *Oncidium panamense* (PAN), and *Rossioglossum ampliatum* (AMP) forward (F) and reverse (R) primer sets were used for one round of amplification

| | |
|---|---|
| MAD1-F | GGAGCTTGTGAAAGTAGCCAAGG |
| MAD1-3R | AATACAAAACACAGGAAGCGGGAG |
| MAD2-F | GAAGACCTAATAAAGGTTGCAAAAC |
| MAD2-3R | CAACTCCCTTCTCTCAATTATGTC |
| MAD3F | TTGGCAGTTGTACAAGGCTCAGGAGG |
| PAN1-F | GCTGGCCGTCTTTCTGCAGC |
| PAN1-2R | CAGTATCTCCCAATCTTTATTTTAATATG |
| PAN2F | TTCGTGTTACCGTGCAGGGTGAAGTTATA |
| PAN3-F | GAAGACCTAATAAAGGTTGCAAAAC |
| PAN3-R | CAGAAGTTTCAGCATCATTTTAACAC |
| AMP1-F | GAGCTTGTGGAAGTAGCCAAGC |
| AMP2-F | GAGCTTGTGAAAGTAGCCAAGG |
| AMP1-2R | TTGAACGTACATCACATTGAATGC |
| AMP3-F | CCATCTAAACGAAAACCTAGTGGT |
| AMP4-F | CCATCTAAACGAAAACCTAGCGGA |
| AMP3-4-F | GAAGACCTAATAAAGGTTGCAAAAC |
| AMP3-4R | AGAAGCAAAGATAAAGCAAAATC |
| AMP3-5F | GACGTCTTTCTGCAGTTGGCAGTTG |
| AMP4-5F | GACGTTTTTCTGCAGCTTGGCAGTTG |
| AMP5R | GATGCAAATTATAAGCACTTGGATGC |

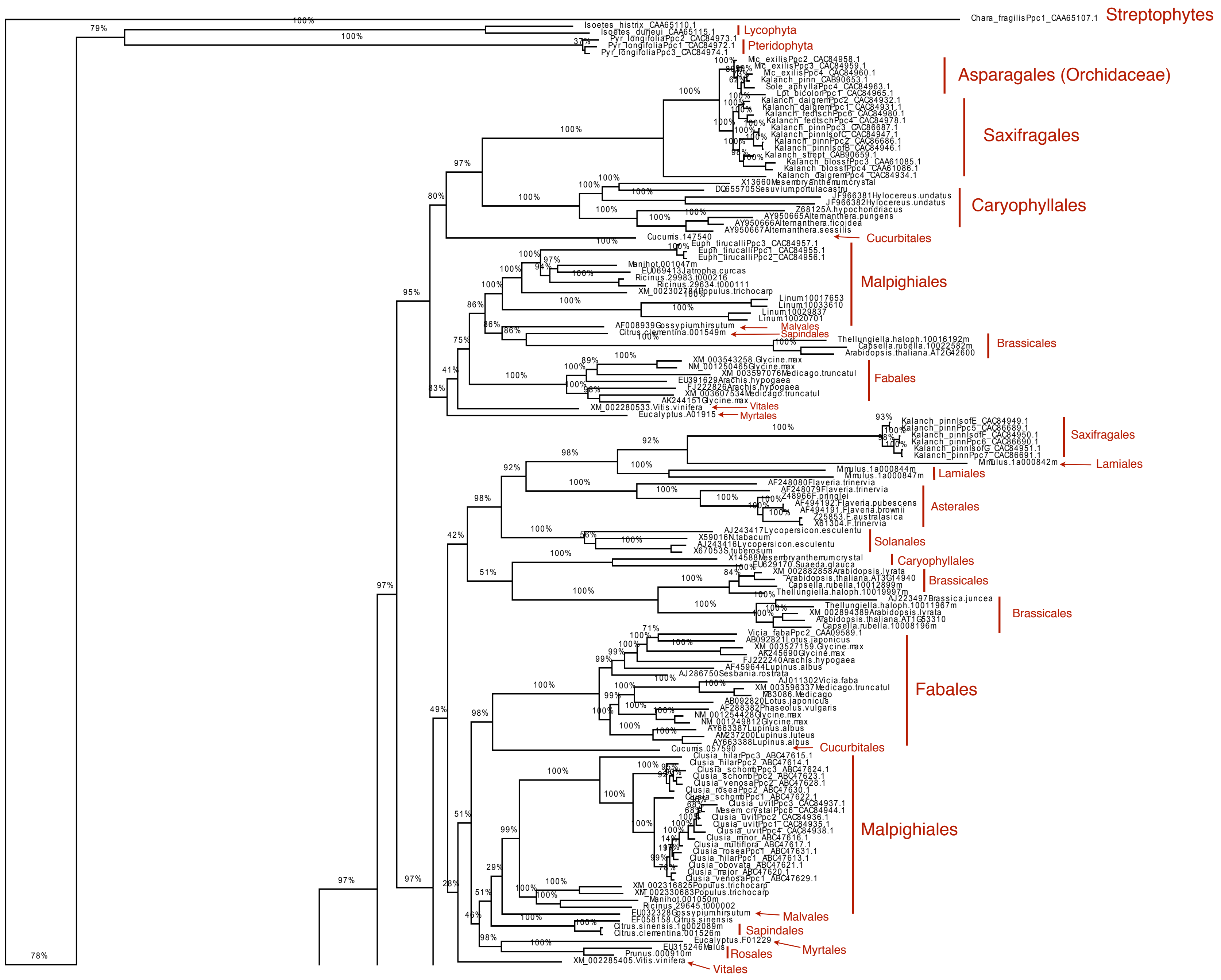
Streptophytes
Chara_fragilisPpc1_CAA65107.1
Isoetes_histrix_CAA65110.1
Isoetes_durieui_CAA65115.1
Lycophyta
Pyr_longifoliaPpc2_CAC84973.1
Pyr_longifoliaPpc1_CAC84972.1
Pyr_longifoliaPpc3_CAC84974.1
Pteridophyta
Asparagales (Orchidaceae)
Mc_exilisPpc2_CAC84958.1
Mc_exilisPpc3_CAC84959.1
Mc_exilisPpc4_CAC84960.1
Kalanch_pinn_CAB90653.1
Sole_aphyllaPpc4_CAC84963.1
Lpt_bicolorPpc1_CAC84965.1
Kalanch_daigremPpc2_CAC84932.1
Kalanch_daigremPpc1_CAC84931.1
Kalanch_fedtschPpc6_CAC84980.1
Kalanch_fedtschPpc4_CAC84978.1
Kalanch_pinnPpc3_CAC86687.1
Kalanch_pinnIsofC_CAC84947.1
Kalanch_pinnPpc2_CAC86686.1
Kalanch_pinnIsofB_CAC84946.1
Kalanch_strept_CAB90659.1
Kalanch_blossfPpc3_CAA61085.1
Kalanch_blossfPpc4_CAA61086.1
Kalanch_daigremPpc4_CAC84934.1
Saxifragales
X13660Mesembryanthemumcrystal
DQ655705Sesuviumportulacastru
JF966381Hylocereus.undatus
JF966382Hylocereus.undatus
Z68125A.hypochondriacus
AY950665Alternanthera.pungens
AY950666Alternanthera.ficoidea
AY950667Alternanthera.sessilis
Caryophyllales
Cucumis.147540
Cucurbitales
Euph_tirucalliPpc3_CAC84957.1
Euph_tirucalliPpc1_CAC84955.1
Euph_tirucalliPpc2_CAC84956.1
Manihot.001047m
EU069413Jatropha.curcas
Ricinus.29983.t000216
Ricinus.29634.t000111
XM_002302764Populus.trichocarp
Linum10017653
Linum10033610
Linum10029837
Linum10020701
Malpighiales
AF008939Gossypiumhirsutum
Malvales
Citrus.clementina.001549m
Sapindales
Thellungiella.haloph.10016192m
Capsella.rubella.10022582m
Arabidopsis.thaliana.AT2G42600
Brassicales
XM_003543258.Glycine.max
NM_001250465Glycine.max
XM_003597076Medicago.truncatul
EU391629Arachis.hypogaea
FJ222826Arachis.hypogaea
XM_003607534Medicago.truncatul
AK244151Glycine.max
Fabales
XM_002280533.Vitis.vinifera
Vitales
Eucalyptus.A01915
Myrtales
Kalanch_pinnIsofE_CAC84949.1
Kalanch_pinnPpc5_CAC86689.1
Kalanch_pinnIsofF_CAC84950.1
Kalanch_pinnPpc6_CAC86690.1
Kalanch_pinnIsofG_CAC84951.1
Kalanch_pinnPpc7_CAC86691.1
Saxifragales
Mimulus.1a000842m
Lamiales
Mimulus.1a000844m
Mimulus.1a000847m
Lamiales
AF248080Flaveria.trinervia
AF248079Flaveria.trinervia
Z48966F.pringlei
AF494192.Flaveria.pubescens
AF494191.Flaveria.brownii
Z25853.F.australasica
X61304.F.trinervia
Asterales
AJ243417Lycopersicon.esculentu
X59016N.tabacum
AJ243416Lycopersicon.esculentu
X67053S.tuberosum
Solanales
X14588Mesembryanthemumcrystal
EU629170.Suaeda.glauca
Caryophyllales
XM_002882858Arabidopsis.lyrata
Arabidopsis.thaliana.AT3G14940
Capsella.rubella.10012899m
Thellungiella.haloph.10019997m
Brassicales
AJ223497Brassica.juncea
Thellungiella.haloph.10011967m
XM_002894389Arabidopsis.lyrata
Arabidopsis.thaliana.AT1G53310
Capsella.rubella.10008196m
Brassicales
Vicia_fabaPpc2_CAA09589.1
AB092821Lotus.japonicus
XM_003527159.Glycine.max
AK245690Glycine.max
FJ222240Arachis.hypogaea
AF459644Lupinus.albus
AJ286750Sesbania.rostrata
AJ011302Vicia.faba
XM_003596337Medicago.truncatul
M83086.Medicago
AB092820Lotus.japonicus
AF288382Phaseolus.vulgaris
NM_001254428Glycine.max
NM_001249812Glycine.max
AY663387Lupinus.albus
AM237200Lupinus.luteus
AY663388Lupinus.albus
Fabales
Cucumis.057590
Cucurbitales
Clusia_hilarPpc3_ABC47615.1
Clusia_hilarPpc2_ABC47614.1
Clusia_schombPpc3_ABC47624.1
Clusia_schombPpc2_ABC47623.1
Clusia_venosaPpc2_ABC47628.1
Clusia_roseaPpc2_ABC47630.1
Clusia_schombPpc1_ABC47622.1
Clusia_uvitPpc3_CAC84937.1
Mesem_crystalPpc6_CAC84944.1
Clusia_uvitPpc2_CAC84936.1
Clusia_uvitPpc1_CAC84935.1
Clusia_uvitPpc4_CAC84938.1
Clusia_minor_ABC47616.1
Clusia_multiflora_ABC47617.1
Clusia_roseaPpc1_ABC47631.1
Clusia_hilarPpc1_ABC47613.1
Clusia_obovata_ABC47621.1
Clusia_major_ABC47620.1
Clusia_venosaPpc1_ABC47629.1
XM_002316825Populus.trichocarp
XM_002330683Populus.trichocarp
Manihot.001050m
Ricinus.29645.t000002
Malpighiales
EU032328Gossypiumhirsutum
Malvales
EF058158.Citrus.sinensis
Citrus.sinensis.1g002089m
Citrus.clementina.001526m
Sapindales
Eucalyptus.F01229
Myrtales
EU315246Malus
Prunus.000910m
Rosales
XM_002285405.Vitis.vinifera
Vitales

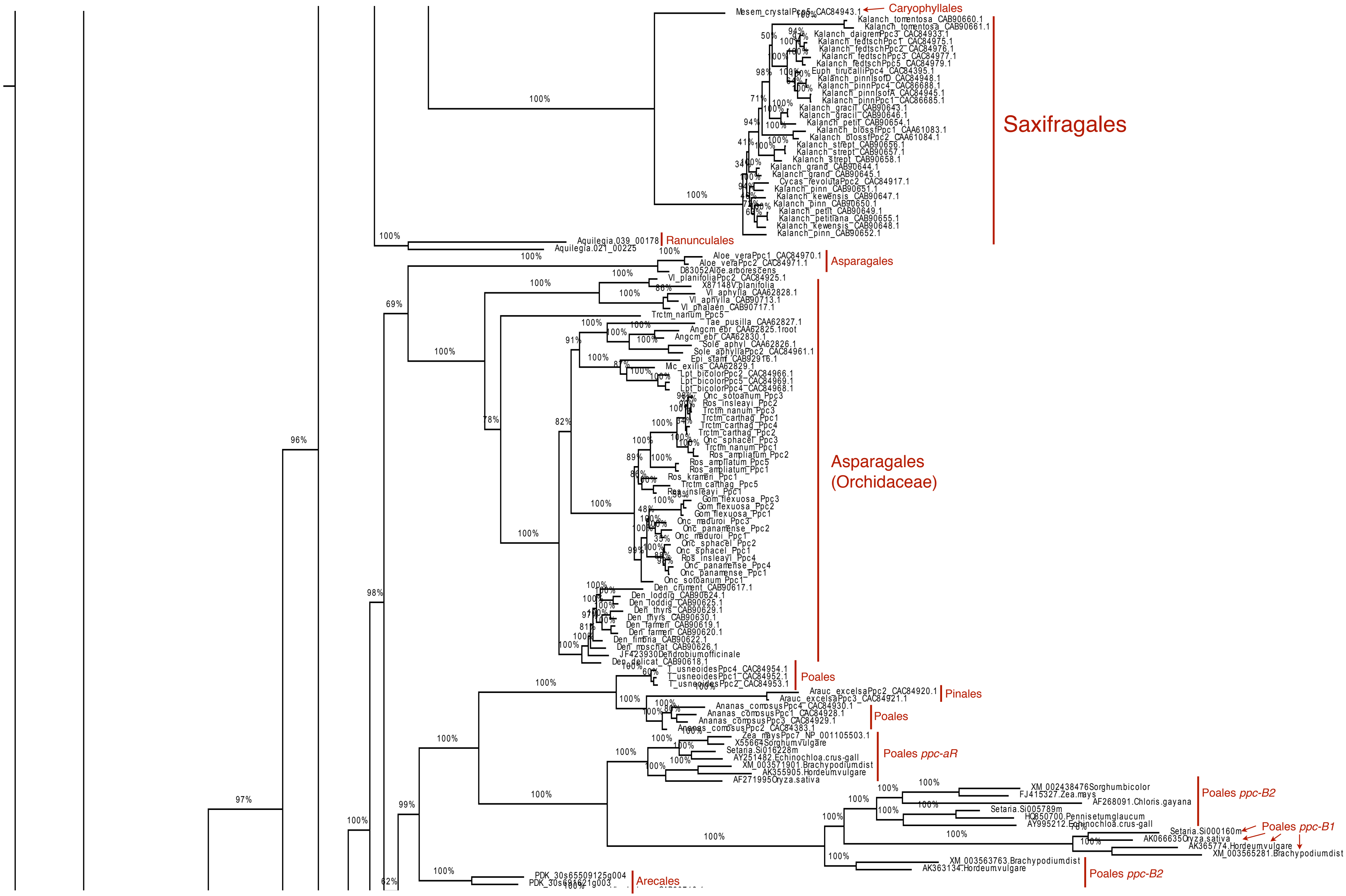
Caryophyllales
Saxifragales
Ranunculales
Asparagales
Asparagales
(Orchidaceae)
Poales
Pinales
Poales
Poales ppc-aR
Poales ppc-B2
Poales ppc-B1
Poales ppc-B2
Arecales

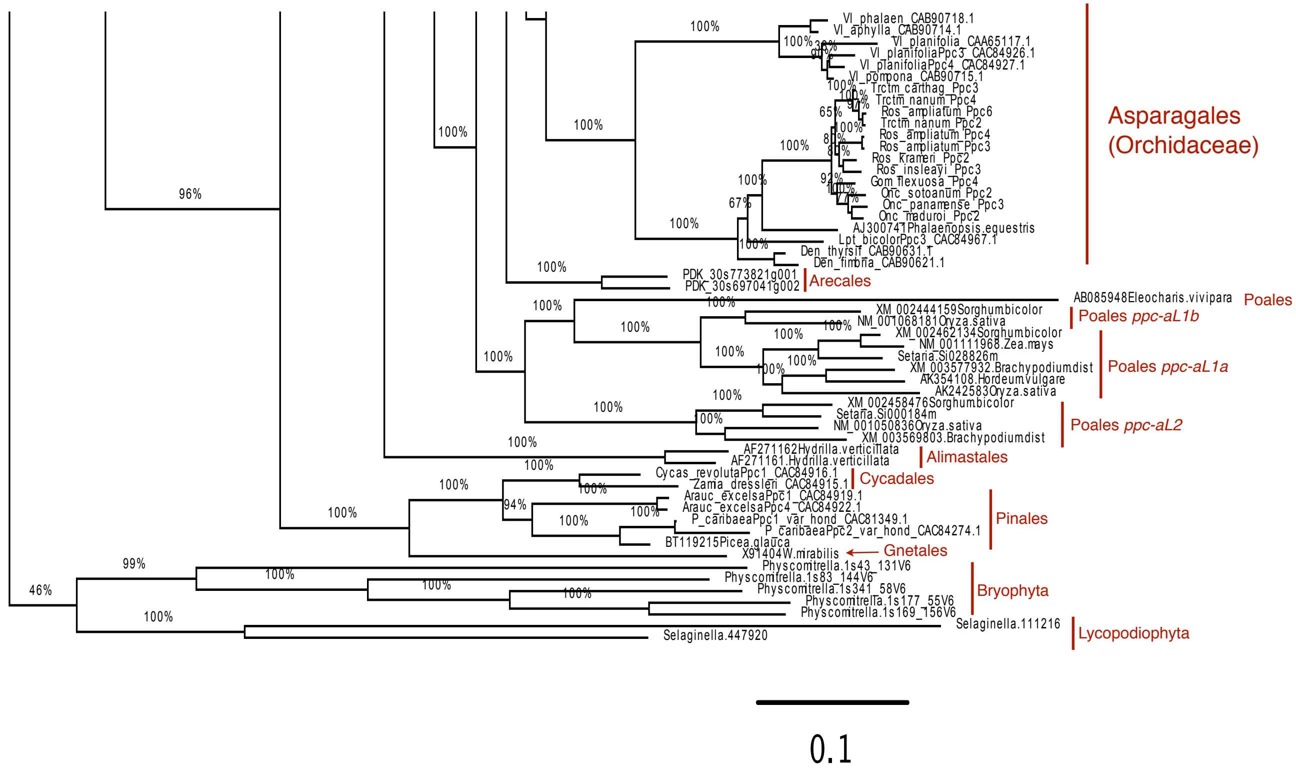


**Fig. S1** Phylogenetic tree of 312 *ppc-1* gene sequences. The tree was obtained through Bayesian analysis.

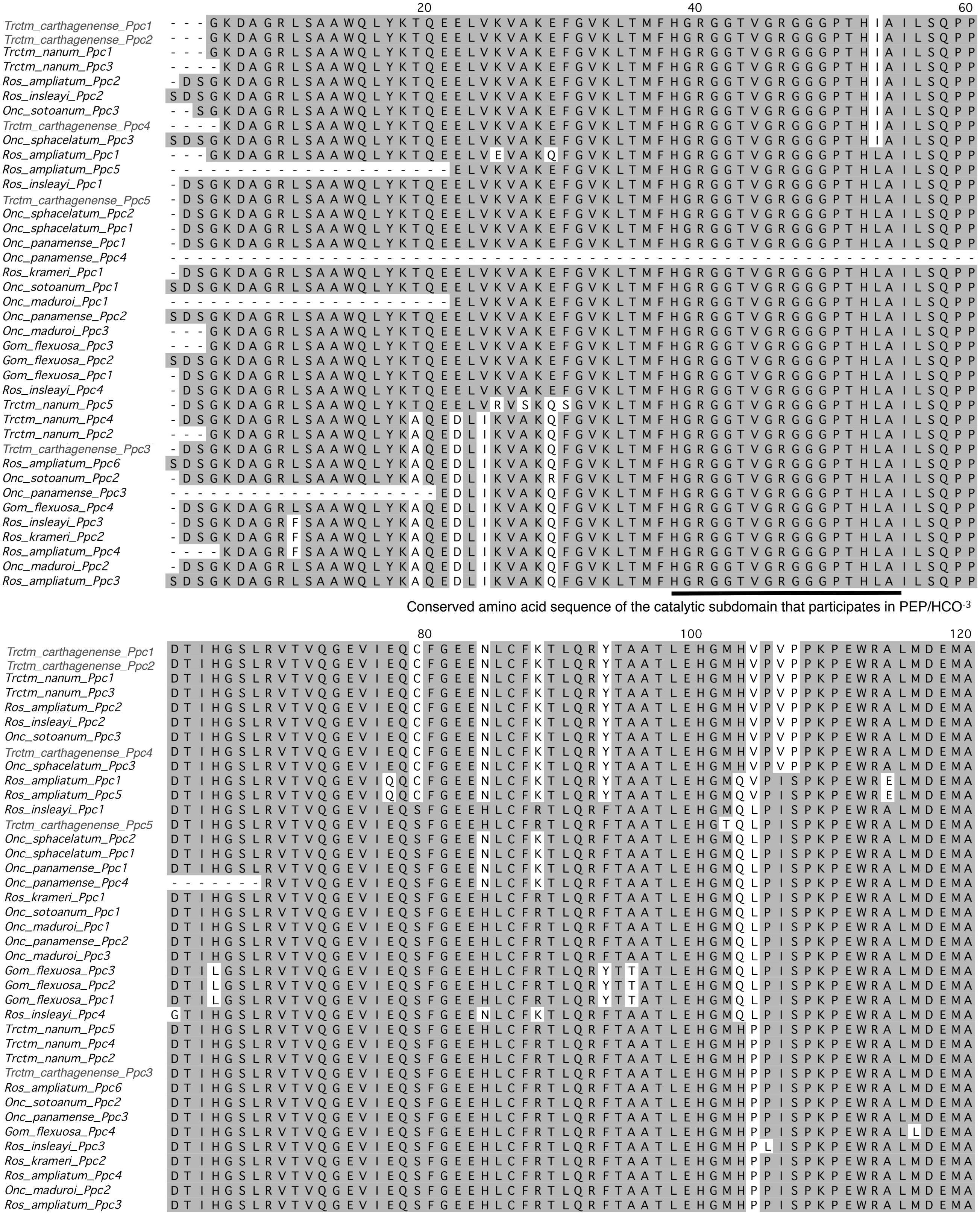
Conserved amino acid sequence of the catalytic subdomain that participates in PEP/HCO-3

```
                                      140                 160                 180
Trctm_carthagenense_Ppc1     IVATNEYRSIVFQEPRFVEYFRLATPELEYGRMNIGSRPSKRKPSGGIESLRAIPWIFAW
Trctm_carthagenense_Ppc2     IVATNEYRSIVFQEPRFVEYFRLATPELEYGRMNIGSRPSKRKPSGGIESLRAIPWIFAW
Trctm_nanum_Ppc1             IVATNEYRSIVFQEPRFVEYFRLATPELEYGRMNIGSRPSKRKPSGGIESLRAIPWIFAW
Trctm_nanum_Ppc3             IVATNEYRSIVFQEPRFVEYFRLATPELEYGRMNIGSRPSKRKPSGGIESLRAIPWIFAW
Ros_ampliatum_Ppc2           IVATNEYRSIVFQEPRFVEYFRLATPELEYGRMNIGSRPSKRKPSGGIESLRAIPWIFAW
Ros_insleayi_Ppc2            IVATNEYRSIVFQEPRFVEYFRLATPELEYGRMNIGSRPSKRKPSGGIESLRAIPWIFAW
Onc_sotoanum_Ppc3            IVATNEYRSIVFQEPRFVEYFRLATPELEYGRMNIGSRPSKRKPSGGIESLRAIPWIFAW
Trctm_carthagenense_Ppc4     IVATNEYRSIVFQEPRFVEYFRLATPELEYGRMNIGSRPSKRKPSGGIESLRAIPWIFAW
Onc_sphacelatum_Ppc3         IVATNEYRSIVFQEPRFVEYFRLATPELEYGRMNIGSRPSKRKPSGGIESLRAIPWIFAW
Ros_ampliatum_Ppc1           IVATNEYRSVVFGEPRFVEYFRLATPELEYGRMNIGSRPSKRKPSGGIESLRAIPWIFAW
Ros_ampliatum_Ppc5           IVATNEYRSVVFGEPRFVEYFRLATPELEYGRMNIGSRPSKRKPSGGIESLRAIPWIFAW
Ros_insleayi_Ppc1            IVATNEYRSIVFQEPRFVEYFRLATPELEYGRMNIGSRPSKRKPSGGIESLRAIPWIFAW
Trctm_carthagenense_Ppc5     IVATNEYRSIVFQEPRFVEYFRLATPELEYGRMNIGSRPSKRKPSGGIESLRAIPWIFAW
Onc_sphacelatum_Ppc2         IVATNEYRSIVFQEPRFVEYFRLATPELEYGRMNIGSRPSKRKPSGGIESPRAIPWIFAW
Onc_sphacelatum_Ppc1         IVATNEYRSIVFQEPRFVEYFRLATPELEYGRMNIGSRPSKRKPSGGIESLRAIPWIFAW
Onc_panamense_Ppc1           IVATNEYRSIVFQEPRFVEYFRLATPELEYGRMNIGSRPSKRKPSGGIESLRAIPWIFAW
Onc_panamense_Ppc4           IVATNEYRSIVFQEPRFVEYFRLATPELEYGRMNIGSRPSKRKPSGGIESLRAIPWIFAW
Ros_krameri_Ppc1             IVATNEYRSIVFEEPRFVEYFRLATPELEYGRMNIGSRPSKRKPSGGIESLRAIPWIFAW
Onc_sotoanum_Ppc1            IVATKEYRSIVFEEPRFVEYFRLATPELEYGRMNIGSRPSKRKPSGGIESLRAIPWIFAW
Onc_maduroi_Ppc1             IVATDEYRSIVFQEPRFVEYFRLATPELEYGRMNIGSRPSKRKPSGGIESLRAIPWIFAW
Onc_panamense_Ppc2           IVATDEYRSIVFQEPRFVEYFRLATPELEYGRMNIGSRPSKRKPSGGIESLRAIPWIFAW
Onc_maduroi_Ppc3             IVATNEYRSIVFQEPRFVEYFRLATPELEYGRMNIGSRPSKRKPSGGIESLRAIPWIFAW
Gom_flexuosa_Ppc3            IVATNEYRSIVFQEPRFVEYFRLATPELEYGRMNIGSRPSKRKPSGGIESLRAIPWIFAW
Gom_flexuosa_Ppc2            IVATNEYRSIVFQEPRFVEYFRLATPELEYGRMNIGSRPSKRKPSGGIESLRAIPWIFAW
Gom_flexuosa_Ppc1            IVATNEYRSIVFQEPRFVEYFRLATPELEYGRMNIGSRPSKRKPSGGIESLRAIPWIFAW
Ros_insleayi_Ppc4            IVATNEYRSIVFQEPRFVEYFRLATPELEYGRMNIGSRPSKRKPSGGIESLRAIPWIFAW
Trctm_nanum_Ppc5             IVATKEYRSVVFQEPGFVEYFRRATPVLEYGRMNIGSRPSKRKPSGGIESLRAIPWIFAW
Trctm_nanum_Ppc4             VVASKEYRSIVFHEPRFVEYFRLATPEMEYGRMNIGSRPSKRKPSGGIESLRAIPWIFAW
Trctm_nanum_Ppc2             VVASKEYRSIVFHEPRFVEYFRLATPEMEYGRMNIGSRPSKRKPSGGIESLRAIPWIFAW
Trctm_carthagenense_Ppc3     VVASKEYRSIVFHEPRFVEYFRLATPEMEYGRMNIGSRPSKRKPSGGIESLRAIPWIFAW
Ros_ampliatum_Ppc6           VVASKEYRSIVFHEPRFVEYFRLATPEMEYGRMNIGSRPSKRKPSGGIESLRAIPWIFAW
Onc_sotoanum_Ppc2            VVATEEYRSIVFHEPRFVEYFRLATPEMEYGRMNIGSRPSKRKPSGGIESLRAIPWIFAW
Onc_panamense_Ppc3           VVATEEYRSIVFHESRFVEYFRLATPEMEYGRMNIGSRPSKRKPSGGIESLRAIPWIFAW
Gom_flexuosa_Ppc4            VVATKEYRSIVFHESRFVEYFRLATPEMEYGRMNIGSRPSKRKPSGGIESLRAIPWIFAW
Ros_insleayi_Ppc3            VVATKEYRSIVFHEPRFVEYFRLATPEMEYGRMNIGSRPSKRKPSGGIESLRAIPWIFAW
Ros_krameri_Ppc2             VVATKEYRSIVFHEPRFVEYFRLATPEMEYGRMNIGSRPSKRKPSGGIESLRAIPWIFAW
Ros_ampliatum_Ppc4           VVATEEYRSIVFHEPRFVEYFRLATPEMEYGRMNIGSRPSKRKPSGGIESLRAIPWIFAW
Onc_maduroi_Ppc2             VVATEEYRSIVFHEPRFVEYFRLATPEMEYGRMNIGSRPSKRKPSGGIESLRAIPWIFAW
Ros_ampliatum_Ppc3           VVATEEYRSIVFHEPRFVEYFRLATPEMEYGRMNIGSRPSKRKPSGGIESLRAIPWIFAW
```

Conserved Ala residue in $C_3$ and CAM, but that changes to Ser in $C_4$ PEPC ●

```
                                               200                 220                 240
Trctm_carthagenense_Ppc1     TQTRFHLPVWLGFGSAFKHAIGKDIKNLQLLRDMYNEWPFFRVTIDLIEMVFTKGNSGIA
Trctm_carthagenense_Ppc2     TQTRFHLPVWLGFGSAFKHAIGKDIKNLQLLRDMYNEWPFFRVTIDLIEMVFTKGNSGIA
Trctm_nanum_Ppc1             TQTRFHLPVWLGFGSAFKHVIEKDIKNLQLLRDMYNEWPFFRVTIDLIEMVFTKGNSGIA
Trctm_nanum_Ppc3             TQTRFHLPVWLGFGSAFKHAIGKDIKNLQLLRDMYNEWPFFRVTIDLIEMVFTKGNSGIA
Ros_ampliatum_Ppc2           TQTRFHLPVWLGFGSAFKHVIEKDIKNLQLLRDMYNEWPFFRVTIDLIEMVFTKGNSGIA
Ros_insleayi_Ppc2            TQTRFHLPVWLGFGSAFKHAIGKDIKNLQLLRDMYNEWPFFRVTIDLIEMVFTKGNSGIA
Onc_sotoanum_Ppc3            TQTRFHLPVWLGFGSAFKHAIGKDIKNLQLLRDMYNEWPFFRVTIDLIEMVFTKGNSGIA
Trctm_carthagenense_Ppc4     TQTRFHLPVWLGFGSAFKHAIGKDIKNLQLLRDMYNEWPFFRVTIDLIEMVFTKGNSGIA
Onc_sphacelatum_Ppc3         TQTRFHLPVWLGFGSAFKHVIEKDIKNLQLLRDMYNEWPFFRVTIDLIEMVFTKGNSGIA
Ros_ampliatum_Ppc1           TQTRFHLPVWLGFGAAFKHVIGKDIKNLSILREMYNEWPFFRVTIDLIEMVFTKGNSRIA
Ros_ampliatum_Ppc5           TQTRFHLPVWLGFGAAFKHVIGKDIKNLSILREMYNEWPFFRVTIDLIEMVFTKGNSRIA
Ros_insleayi_Ppc1            TQTRFHLPVWLGFGAAFKHVIGKDIKNLHILREMYNEWPFFRVTIDLIEMVFTKGSSGIA
Trctm_carthagenense_Ppc5     TQTRFHLPVWLGFGAAFKHVIGKDIKNLHILREMYNEWPFFRVTIDLIEMVFTKGSSGIA
Onc_sphacelatum_Ppc2         TQTRFHLPVWLGSGAAFKHVIGKDIKNLHLLQEMYNEWPFFRVTIDLIEMVFTKGSPGIA
Onc_sphacelatum_Ppc1         TQTRFHLPVWLGFGAAFKHVIGKDIKNLHLLQEMYNEWPFFRVTIDLIEMVFTKGSPGIA
Onc_panamense_Ppc1           TQTRFHLPVWLGFGAAFKHVIGKDIKNLHLLQEMYNEWPFFRVTIDLIEMVFTKGSPGIA
Onc_panamense_Ppc4           TQTRFHLPVWLGFGAAFKHVIGKDIKNLHLLQEMYNEWPFFRVTIDLIEMVFTKGSPGIA
Ros_krameri_Ppc1             TQTRFHLPVWLGFGAAFKHVIGKDIKNLHILREMYNEWPFFRVTIDLIEMVFTKGSTGIA
Onc_sotoanum_Ppc1            TQTRFHLPVWLGFGAAFKHVIGKDIKNLHILQEMYNEWPFFRVTIDLIEMVFTKGSPGIA
Onc_maduroi_Ppc1             TQTRFHLPVWLGFGAAFKHVIGKDIKNLHILREMYNEWPFFRVTIDLIEMVFTKGSPGIA
Onc_panamense_Ppc2           TQTRFHLPVWLGFGAAFKHVIGKDIKNLHILREMYNEWPFFRVTIDLIEMVFTKGSPGIA
Onc_maduroi_Ppc3             TQTRFHLPVWLGFGAAFKHVIGKDIKNLHILREMYNEWPFFRVTIDLIEMVFTKGSPGIA
Gom_flexuosa_Ppc3            TQTRSHLSVWLGFGGAFKHVIGKDIKNLQILREMYNEWPFFRVTIDLIEMVFTKGSPGIA
Gom_flexuosa_Ppc2            TQTRFHLPVWLGFGGAFKHVIGKDIKNLQILREMYNEWPFFRVTIDLIEMVFTKGSPGIA
Gom_flexuosa_Ppc1            TQTRFHLPVWLGFGGAFKHVIGKDIKNLQILREMYNEWPFFRVTIDLIEMVFTKGSPGIA
Ros_insleayi_Ppc4            TQTRFHLPVWLGFGAAFKHVIGKDIKNLHLLQEMYNEWPFFRVTIDLIEMVFTKGSPGIA
Trctm_nanum_Ppc5             TQTRFHLPVWLGFGAAFKHVMEKDIRNFHILREMYNEWPFFRVTIDLVEMVFAKGSPCIA
Trctm_nanum_Ppc4             TQTRFHLPVWLGFGTAFKHAIEKDIKNLHMLREMYNEWPFFRVTIDLVEMVFAKGDPGIA
Trctm_nanum_Ppc2             TQTRFHLPVWLGFGTAFKHAIEKDIKNLHMLREMYNEWPFFRVTIDLVEMVFAKGDPGIA
Trctm_carthagenense_Ppc3     TQTRFHLPVWLGFGTAFKHAIEKDIKNLHMLREMYNEWPFFRVTIDLVEMVFAKGDPGIA
Ros_ampliatum_Ppc6           TQTRFHLPVWLGFGTAFKHAIEKDIKNLHMLREMYNEWPFFRVTIDLVEMVFAKGDPGIA
Onc_sotoanum_Ppc2            TQTRFHLPVWLGFGTAFKHAIEKDIKNLHMLREMYNEWPFFRVTIDLVEMVFAKGDPGIA
Onc_panamense_Ppc3           TQTRFHLPVWLGFGTAFKHAIEKDIKNLQMLREMYNEWPFFRVTIDLVEMVFAKGDPGIA
Gom_flexuosa_Ppc4            TQTRFHLPVWLGFGTAFKHAIEKDIKNLHMLREMYNEWPFFRVTIDLVEMVFAKGDPGIA
Ros_insleayi_Ppc3            TQTRFHLPVWLGFGTAFKHAIEKDIKNLHMLREMYNEWPFFRVSIDLVEMVFAKGDPGIA
Ros_krameri_Ppc2             TQTRFHLPVWLGFGTAFKHAIEKDIKNLHMLREMYNEWPFFRVSIDLVEMVFAKGDPGIA
Ros_ampliatum_Ppc4           TQTRFHLPVWLGFGTAFKHAIEKDIKNLHMLREMYNEWPFFRVSIDLVEMVFAKGDPGIA
Onc_maduroi_Ppc2             TQTRFHLPVWLGFGTAFKHAIEKDIKNLHMLREMYNEWPFFRVTIDLVEMVFAKGDPGIA
Ros_ampliatum_Ppc3           TQTRFHLPVWLGFGTAFKHAIEKDIKNLHMLREMYNEWPFFRVSIDLVEMVFAKGDPGIA
```

```
                                                   260                 280                 300
Trctm_carthagenense_Ppc1    ALYDKLLVSEDLLPFGEQLRANYEETQRLLLQVAGHKVLLEGDPYLKQRLRLRYPYITTL
Trctm_carthagenense_Ppc2    ALYDKLLVSEDLLPFGEQLRANYEETQRLLLQVAGHKVLLEGDPYLKQRLRLRYPYITTL
Trctm_nanum_Ppc1            ALYDKLLVSEDLLPFGEQLRANYEETQRLLLQVAGHKVLLEGDPYLKQRLRLRYPYITTL
Trctm_nanum_Ppc3            ALYDKLLVSEDLLPFGEQLRANYEETQRLLLQVAGHKVLLEGDPYLKQRLRLRYPYITTL
Ros_ampliatum_Ppc2          ALYDKLLVSEDLLPFGEQLRANYEETQRLLLQVAGHKVLLEGDPYLKQRLRLRYPYITTL
Ros_insleayi_Ppc2           ALYDKLLVSEDLLPFGEQLRANYEETQRLLLQVAGHKVLLEGDPYLKQRLRLRYPYITTL
Onc_sotoanum_Ppc3           ALYDKLLVSEDLLPFGEQLRANYEETQRLLLQVAGHKVLLEGDPYLKQRLRLRYPYITTL
Trctm_carthagenense_Ppc4    ALYDKLLVSEDLLPFGEQLRANYEETQRLLLQVAGHKVLLEGDPYLKQRLRLRYPYITTL
Onc_sphacelatum_Ppc3        ALYDKLLVSEDLLPFGEQLRANYEETQRLLLQVAGHKVLLEGDPYLKQRLRLRYPYITTL
Ros_ampliatum_Ppc1          ALYDKLLVSDDLLPFGEQLRANYEETQRLLLQVAGHKVLLEGDPYLKQRLKLRYPYITTL
Ros_ampliatum_Ppc5          ALYDKLLVSDDLLPFGEQLRANYEETQRLLLQVAGHKVLLEGDPYLKQRLKLRYPYITTL
Ros_insleayi_Ppc1           ALYDKLLVSDDLLPFGEQLRANYEETKGLLLQVAGHKILLEGDPYLKQRLRLRYPYITTL
Trctm_carthagenense_Ppc5    ALYDKLLVSDDLLPFGEQLRANYEETKGLLLQVAGHKILLEGDPYLKQRLRLRYPYITTL
Onc_sphacelatum_Ppc2        ALYDKLLVSDDLLPFGEQLRANYEETKRLLLQVAGHKVLLEGDPYLKQRLRLRYPYITTL
Onc_sphacelatum_Ppc1        ALYDKLLVSDDLLPFGEQLRANYEETKRLLLQVAGHKVLLEGDPYLKQRLRLRYPYITTL
Onc_panamense_Ppc1          ALYDKLLVSDDLLPFGEQLRANYEETKRLLLQVAGHKVLLEGDPYLKQRLRLRYPYITTL
Onc_panamense_Ppc4          ALYDKLLVSDDLLPFGEQLRANYEETKRLLLQVAGHKVLLEGDPYLKQRLRLRYPYITTL
Ros_krameri_Ppc1            ALYDKLLVSDDLLPFGEQLRANYEETKRLLLQVAGHKVLLEGDPYLKQRLRLRYPYITTL
Onc_sotoanum_Ppc1           ALYDKLLVSDDLLPFGEQLRANYEETKRLLLQVAGHKVLLEGDPYLKQRLRLRYPYITTL
Onc_maduroi_Ppc1            ALYDKLLVSDDLLPFGEQLRANYEETKRLLLQVAGHKVLLEGDPYLKQRLRLRYPYITTL
Onc_panamense_Ppc2          ALYDKLLVSDDLLPFGEQLRANYEETKRLLLQVAGHKVLLEGDPYLKQRLRLRYPYITTL
Onc_maduroi_Ppc3            ALYDKLLVSDDLLPFGEQLRANYEETKRLLLQVAGHKVLLEGDPYLKQRLRLRYPYITTL
Gom_flexuosa_Ppc3           ALYDKLLVSDDLLPFGEQLRANYIETTDLLLQVAGHKVLLEGDPYLKQRLRLRYPYITTL
Gom_flexuosa_Ppc2           ALYDKLLVSDDLLPFGEQLRANYIETTDLLLQVAGHKVLLEGDPYLKQRLRLRYPYITTL
Gom_flexuosa_Ppc1           ALYDKLLVSDDLLPFGEQLRANYIETTDLLLQVAGHKVLLEGDPYLKQRLRLRYPYITTL
Ros_insleayi_Ppc4           ALYDKLLVSDDLLPFGEQLRANYEETKRLLLQVAGHKVLLEGDPYLKQRLRLRYPYITTL
Trctm_nanum_Ppc5            ALYDKLLVSSDLLPFGDQLRANYEETKRLLLQVAGHKDILEEDPYLKQRLRLRDPYITTL
Trctm_nanum_Ppc4            ELYDKLLVSDDLWSFGQRLRANYEETKNHLLQVAGHKDLLEGDPYLKQRLRLRHSYITTL
Trctm_nanum_Ppc2            ELYDKLLVSDDLWSFGQRLRANYEETKNHLLQVAGHKDLLEGDPYLKQRLRLRHSYITTL
Trctm_carthagenense_Ppc3    ELYDKLLVSDDLWSFGQRLRANYEETKDHLLQVAGHKDLLEGDPYLKQRLRLRHSYITTL
Ros_ampliatum_Ppc6          ELYDKLLVSDDLWSFGQRLRANYEETKNHLLQVAGHKDLLEGDPYLKQRLRLRHSYITTL
Onc_sotoanum_Ppc2           ELYDKLLVSDDLWSFGQRLRANYEETKSLLLQVAGHKDLLEGDPYLKQRLRLRHSYITTL
Onc_panamense_Ppc3          ELYDKLLVSDDLWSFGQRLRANYEETKNLLRVAGHKDLLEGDPYLKQRLRLRHSYITTL
Gom_flexuosa_Ppc4           ELYDKLLVSDDLWSFGQRLRASYEETKDLLRVAGHKDLLEGDPYLKQRLRLRHSYITTL
Ros_insleayi_Ppc3           ELYDKLLVSDDLWSFGQRLRANYEETKNLLRVAGHKDLLEGDPYLKQRLRLRDSYITTL
Ros_krameri_Ppc2            ELYDKLLVSDDLWSFGQRLRANYEETKNLLRVAGHKDLLEGDPYLKQRLRLRHSYITTL
Ros_ampliatum_Ppc4          ELYDKLLVSDDLWSFGQRLRANYEETKNLLLQVAGHKDLLEGDPYLKQRLRLRHSYITTL
Onc_maduroi_Ppc2            ELYDKLLVSDDLWSFGQRLRANYEETKNLLRVAGHKDLLEGDPYLKQTLRLRHSYITTL
Ros_ampliatum_Ppc3          ELYDKLLVSDDLWSFGQRLRANYEETKNLLLQVAGHKDLLEGDPYLKQRLRLRHSYITTL
```

```
                                                   320                 340                 360
Trctm_carthagenense_Ppc1    NVCQAYTLKRIRDPSNGQDAKPDPSKEINNSNKTAAELVNLNPTSEYAPGMEDTLILTMK
Trctm_carthagenense_Ppc2    NVCQAYTLKRIRDPSNGQDAKPDPSKEINNSNKTAAELVKLNPTSEYAPGMEDTLILTMK
Trctm_nanum_Ppc1            NVCQAYTLKRIRDPSDGQDAKPDPSKEINNSSKTAAELVKLNPTSEYAPGLEDTLILTMK
Trctm_nanum_Ppc3            NVCQAYTLKRIRDPSNGQDAKPDPSKEINNSNKTAAELVNLNPTSEYAPGMEDTLILTMK
Ros_ampliatum_Ppc2          NVCQAYTLKRIRDPSDGQDAKPDPSKEINNSSKTAAELVKLNPTSEYAPGLEDTLILTMK
Ros_insleayi_Ppc2           NVCQAYTLKRIRDPSNGQDAKPDPSKEINNSNKTAAELVNLNPTSEYAPGMEDTLILTMK
Onc_sotoanum_Ppc3           NVCQAYTLKRIRDPSNGQDAKPDPSKEINNSNKTAAELVNLNPTSEYAPGMEDTLILTMK
Trctm_carthagenense_Ppc4    NVCQAYTLKRIRDPSNGQDAKPDPSKEINNSNKTAAELVKLNPTSEYAPGMEDTLILTMK
Onc_sphacelatum_Ppc3        NVCQAYTLKRIRDPSDGQDAKPDPSKEINNSSKTAAELVKLNPTSEYAPGLEDTLILTMK
Ros_ampliatum_Ppc1          NVCQACTLKRIRDPSNGQTTEPDPSKEIKNSNKPAAELVNLNPTSEYAPGLEDTLILTMK
Ros_ampliatum_Ppc5          NVCQACTLKRIRDPSNGQTTEPDPSKEIKNSNKPAAELVNLNPTSEYAPGLEDTLILTMK
Ros_insleayi_Ppc1           NVCQAYTLKRIRDPSDRQTTKPDPSKEIKNSNKPAAELVKLNPASEYAPGLEDTLILTMK
Trctm_carthagenense_Ppc5    NVCQAYTLKRIRDPSDGQDAKPDPSKEINNSSKTAAELVKLNPTSEYAPGLEDTLILTMK
Onc_sphacelatum_Ppc2        NVCQAYALKRIRDPSDHGAAKPDPSKEIKNSNKPAAELVKLNPTSEYAPGLEDTLIITMK
Onc_sphacelatum_Ppc1        NVCQAYALKRIRDPSDHGTAKPDPSKEIKNSNKPAAELVKLNPTSEYAPGLEDTLIITMK
Onc_panamense_Ppc1          NVCQAYALKRIRDPSDHGTAKPDPSKEIKNSNKPAAELVKLNPTSEYAPGLEDTLIITMK
Onc_panamense_Ppc4          NVCQAYALKRIRDPSDHGTAKPDPSKEIKNSNKPAAELVKLNPTSEYAPGLEDTLIITMK
Ros_krameri_Ppc1            NVCQAYTLKRIRDPSDRQAAKPDPSKEMKNSNKPAAELVKLNPTSEYAPGLEDTLILTMK
Onc_sotoanum_Ppc1           NVCQAYTLKRIRDPSDHRTAKPDPSKEIKNSNKPAAELVKLNPTSEYAPGLEDTLILTMK
Onc_maduroi_Ppc1            NVCQAYTLKRIRDPSDHRTAKPDPSKEIKNSNKPAAELVKLNPTSEYAPGLEDTLILTMK
Onc_panamense_Ppc2          NVCQAYTLKRIRDPSDHRTSKPDPSKEIKNSNKPAAELVKLNPTSEYAPGLEDTLILTMK
Onc_maduroi_Ppc3            NVCQAYTLKRIRDPSDHRTAKPDPSKEIKNSNKPAAELVKLNPTSEYAPGLEDTLILTMK
Gom_flexuosa_Ppc3           NVCQAYTLKRIRDPSDHQTDKPDPSKEIKNSNKPAAELVKLNPTSEYAPGLEGTLILTMK
Gom_flexuosa_Ppc2           NVCQAYTLKRIRDPSDHQTDKPDPSKEIKNSNKPAAELVKLNPTSEYAPGLEDTLILTMK
Gom_flexuosa_Ppc1           NVCQAYTLKRIRDPSDHPTDKPDPSKEIKNSNKPAAELVKLNPTSEYAPGLEDTLILTMK
Ros_insleayi_Ppc4           NVCQAYALKRIRDPSDHGTAKPDPSKEIKNSNKPAAELVKLNPTSEYAPGLEDTLIITMK
Trctm_nanum_Ppc5            NVCQAYTLKRIRDPNYHVTARPHLSKEILTSNKSAAELVKLNPTSEYAPGLEDTLILTMK
Trctm_nanum_Ppc4            NVCQAFTLKRIRDPNFLVNVRPHISKEIIDSNKPAAELVKLNPTSEYAPGLEDTLILTMK
Trctm_nanum_Ppc2            NVCQAFTLKRIRDPNFLVNVRPHISKEIIDSNKPAAELVKLNPTSEYAPGLEDTLILTMK
Trctm_carthagenense_Ppc3    NVCQAFTLKRIRDPNFLVNVRPHISKEIMDSNKPAAELVKLNPTSEYAPGLEDTLILTMK
Ros_ampliatum_Ppc6          NVCQAFTLKRIRDPNFLVNVRPHISKEIIDSNKPAAELVKLNPTSEYAPGLEDTLILTMK
Onc_sotoanum_Ppc2           NVCQAFTLKRIRDPNFLVNVRPHISKEIMDSNKPAAELVKLNPTSEYAPGLEDTLILTMK
Onc_panamense_Ppc3          NVCQAFTLKRIRDPNFLVNVRPHISKEIMDSNKPAAELVKLNPTSEYAPGLEDTLILTMK
Gom_flexuosa_Ppc4           NVCQAFTLKRIRDPNFLVNVRPHISREIMDSNKQAAELVKLNPTSEYAPGLEDTLILTMK
Ros_insleayi_Ppc3           NVCQAFTLKRIRDPNFLVNVRPHISKEIMDSNKPAAELVKLNPTSEYAPGLEDTLILTMK
Ros_krameri_Ppc2            NVCQAFTLKRIRDPNFLVNVRPHISKEIMDSNKPAAELVKLNPTSEYAPGLEDTLILTMK
Ros_ampliatum_Ppc4          NVCQAFTLKRIRDPNFLVNVRPHISKEIMDSNKPAAELVKLNPTSEYAPGLEDTLILTMK
Onc_maduroi_Ppc2            NVCQAFTLKRIRDPNFHVNVRPHISKEIMDSNKPAAELVKLNPTSEYAPGLEDTLILTMK
Ros_ampliatum_Ppc3          NVCQAFTLKRIRDPNFLVNVRPHISKEIMDSNKPAAELVKLNPTSEYAPGLEDTLILTMK
```

```
                                           380                 400                 420
Trctm_carthagenense_Ppc1  GIA---------------------------------------------------------
Trctm_carthagenense_Ppc2  GIA---------------------------------------------------------
Trctm_nanum_Ppc1          GIAA--------------------------------------------------------
Trctm_nanum_Ppc3          GI----------------------------------------------------------
Ros_ampliatum_Ppc2        GIA---------------------------------------------------------
Ros_insleayi_Ppc2         GI----------------------------------------------------------
Onc_sotoanum_Ppc3         GI----------------------------------------------------------
Trctm_carthagenense_Ppc4  GI----------------------------------------------------------
Onc_sphacelatum_Ppc3      GIA---------------------------------------------------------
Ros_ampliatum_Ppc1        GIAAGLQNTGISSSCPLSLNFKN-MYTVIPPHVYCLYVFLSTVYLWMYQVLYDSMDIYLP
Ros_ampliatum_Ppc5        GIAAGLQNTGISSSCPLSLNFKN-MYTVIPPHVYCLYVFLSTVYLWMYQVLYDSMDIYLP
Ros_insleayi_Ppc1         GI----------------------------------------------------------
Trctm_carthagenense_Ppc5  GIA---------------------------------------------------------
Onc_sphacelatum_Ppc2      GIA---------------------------------------------------------
Onc_sphacelatum_Ppc1      GI----------------------------------------------------------
Onc_panamense_Ppc1        GIAAGLQNTGISSSWLLSLNLEICVYLLMCIAYMYLYQLLYLWTYPVRVILRQKYGYLSP
Onc_panamense_Ppc4        GIAAGLQNTGISSSWLLSLNLEICVYLLMCIAYMYLYQLLYLWTYPVRVILRQKYGYLSP
Ros_krameri_Ppc1          GI----------------------------------------------------------
Onc_sotoanum_Ppc1         GI----------------------------------------------------------
Onc_maduroi_Ppc1          GIAAGLQNTGISSSWLLSLNFRN-MCMIIPPHVYCLYVFISTLMISLDVVPSEGYIETEV
Onc_panamense_Ppc2        GIAAGLQNTGISSSWLLSLNFGN-MCMIIPPHVYCLYVFISTLMISLDVVSSEGYIETEV
Onc_maduroi_Ppc3          GIAAGLQNTGISSSWLLSLNFTN-MCMIIPPHVYCLYVFISTLMISLDVVPSEGYIETEV
Gom_flexuosa_Ppc3         GIA---------------------------------------------------------
Gom_flexuosa_Ppc2         GI----------------------------------------------------------
Gom_flexuosa_Ppc1         GI----------------------------------------------------------
Ros_insleayi_Ppc4         GIA---------------------------------------------------------
Trctm_nanum_Ppc5          GIA---------------------------------------------------------
Trctm_nanum_Ppc4          GIA---------------------------------------------------------
Trctm_nanum_Ppc2          GIA---------------------------------------------------------
Trctm_carthagenense_Ppc3  GI----------------------------------------------------------
Ros_ampliatum_Ppc6        GIA---------------------------------------------------------
Onc_sotoanum_Ppc2         GI----------------------------------------------------------
Onc_panamense_Ppc3        GIAAGMQNTG-----------PS-ILCFLSIFCIFKLNLFAFVYFELREVFSPQFAVNLK
Gom_flexuosa_Ppc4         GIA---------------------------------------------------------
Ros_insleayi_Ppc3         GI----------------------------------------------------------
Ros_krameri_Ppc2          GI----------------------------------------------------------
Ros_ampliatum_Ppc4        GIAAGMQNTG-----------PS-IYAFN---YQFDFVFLSKTCLIPSILNCVRFSIPNL
Onc_maduroi_Ppc2          GIAAGMQNTG-----------PS-IYAFN---CQFDFVFLS-TCLLSSILNYVRFSVLNL
Ros_ampliatum_Ppc3        GIAAGMQNTG-----------PS-IYAFN---YQFDFVFLSKTCLIPSILNCVRFSIPNL
```

```
                                           440                 460                 480
Trctm_carthagenense_Ppc1  -------------------------------------
Trctm_carthagenense_Ppc2  -------------------------------------
Trctm_nanum_Ppc1          -------------------------------------
Trctm_nanum_Ppc3          -------------------------------------
Ros_ampliatum_Ppc2        -------------------------------------
Ros_insleayi_Ppc2         -------------------------------------
Onc_sotoanum_Ppc3         -------------------------------------
Trctm_carthagenense_Ppc4  -------------------------------------
Onc_sphacelatum_Ppc3      -------------------------------------
Ros_ampliatum_Ppc1        LPLFCYTVSFIIKRLGDTAFNVMYVQ-----------
Ros_ampliatum_Ppc5        LPLFCYTVSFIIKRLGDTAFNVMYVQ-----------
Ros_insleayi_Ppc1         -------------------------------------
Trctm_carthagenense_Ppc5  -------------------------------------
Onc_sphacelatum_Ppc2      -------------------------------------
Onc_sphacelatum_Ppc1      -------------------------------------
Onc_panamense_Ppc1        ASYVLYYCIFHIKIKIGRY------------------
Onc_panamense_Ppc4        ASYVLYYCIFHIKIKIGRY------------------
Ros_krameri_Ppc1          -------------------------------------
Onc_sotoanum_Ppc1         -------------------------------------
Onc_maduroi_Ppc1          WIFISRFLCFV--------------------------
Onc_panamense_Ppc2        WIFISRFLCFVLLYLSYNKDWEIL-------------
Onc_maduroi_Ppc3          WIFISRFLCFV--------------------------
Gom_flexuosa_Ppc3         -------------------------------------
Gom_flexuosa_Ppc2         -------------------------------------
Gom_flexuosa_Ppc1         -------------------------------------
Ros_insleayi_Ppc4         -------------------------------------
Trctm_nanum_Ppc5          -------------------------------------
Trctm_nanum_Ppc4          -------------------------------------
Trctm_nanum_Ppc2          -------------------------------------
Trctm_carthagenense_Ppc3  -------------------------------------
Ros_ampliatum_Ppc6        -------------------------------------
Onc_sotoanum_Ppc2         -------------------------------------
Onc_panamense_Ppc3        IDILEIIESRELCNDAETS------------------
Gom_flexuosa_Ppc4         -------------------------------------
Ros_insleayi_Ppc3         -------------------------------------
Ros_krameri_Ppc2          -------------------------------------
Ros_ampliatum_Ppc4        HSIWRLIYKLREVSCVKMMLKLLSECVFLMILLYLCF
Onc_maduroi_Ppc2          H--IKFIYTLREGS-----------------------
Ros_ampliatum_Ppc3        HSIWRLIYKLREVSCVKMMLKLLSECVFLMILLYLCF
```

**Fig. S2** Protein sequence alignment for 28 PEPC isoforms, and 11 PEPC isoforms recovered by RACE for 10 species of the subtribe Oncidiinae
